# The Relation of Personality and Intelligence—What Can the Brunswik Symmetry Principle Tell Us?

**DOI:** 10.3390/jintelligence6030030

**Published:** 2018-07-03

**Authors:** André Kretzschmar, Marion Spengler, Anna-Lena Schubert, Ricarda Steinmayr, Matthias Ziegler

**Affiliations:** 1 Hector Research Institute of Education Sciences and Psychology, University of Tübingen, Europastraße 6, 72072 Tübingen, Germany; marion.spengler@uni-tuebingen.de; 2 Institute of Psychology, Heidelberg University, Hauptstrasse 47-51, D-69117 Heidelberg, Germany; anna-lena.schubert@psychologie.uni-heidelberg.de; 3 Department of Psychology, Technical University Dortmund, Emil-Figge-Straße 50, 44227 Dortmund, Germany; ricarda.steinmayr@tu-dortmund.de; 4 Institute of Psychology, Humboldt-Universität zu Berlin, Rudower Chaussee 18, 12489 Berlin, Germany; zieglema@hu-berlin.de

**Keywords:** personality, Big Five, intelligence, Berlin Intelligence Structure model, Brunswik Symmetry, bandwidth fidelity, integrative data analysis

## Abstract

Personality and intelligence are defined as hierarchical constructs, ranging from broad *g*-factors to (domain-)specific constructs. The present study investigated whether different combinations of hierarchical levels lead to different personality-intelligence correlations. Based on the integrative data analysis approach, we combined a total of five data sets. The focus of the first study (*N* = 682) was an elaborated measurement of personality (NEO-PI-R), which was applied with a relatively short intelligence test (Intelligence Structure Test 2000 R). In the second study (*N* = 413), a comprehensive measurement of intelligence (Berlin Intelligence Structure test) was used with a shorter personality questionnaire (NEO-FFI). In line with the Brunswik symmetry principle, the findings emphasize that personality-intelligence correlations varied greatly across the hierarchical levels of constructs considered in the analysis. On average, Openness showed the largest relation with intelligence. We recommend for future studies to investigate personality-intelligence relations at more fine-grained levels based on elaborated measurements of both personality and intelligence.

## 1. Introduction

The relation between intelligence and personality is not only of high theoretical but also practical importance as personality and intelligence tests are often both applied in selection contexts. Knowing their relation gives a hint on the incremental and combined validity of both constructs when predicting important criteria such as job performance. Furthermore, the relation between constructs is important for building psychological theories which aim to understand the complexity of human nature. One popular way of doing this is to derive and test trait taxonomies describing important areas of personality and ability. In the field of intelligence research, the majority of contemporary models (i.e., taxonomies) define intelligence as a hierarchical, multidimensional construct instead of a simple, unidimensional construct. For example, based on the comprehensive and integrative Berlin Intelligence Structure (BIS) model [1,2], three hierarchical levels can be distinguished: general intelligence (*g*) at the top, four operative abilities (e.g., fluid reasoning^1^, perceptual speed) and three content-related abilities (e.g., verbal intelligence) at the next lower level and 12 more specific abilities (e.g., verbal reasoning) at the lowest level. Taxonomies focusing on personality traits are conceptualized in a similar manner. For example, in the currently predominant Five Factor Model (FFM) [3], personality is represented with five broad domains (Big Five; e.g., Openness to Experiences) and several narrow facets within each domain (e.g., Openness to Values or Openness to Aesthetics). In recent years, the Pyramidal Model of personality [4,5] was proposed as an extension of the FFM that additionally includes higher-order factors of personality. The idea of higher order factors above the Big Five has been introduced by Digman [6] who proposed the two higher order factors alpha (Neuroticism, Agreeableness and Conscientiousness) and beta (Extraversion and Openness), later called Stability and Plasticity (Big Two) by DeYoung et al. [7]. An alternative higher order conceptualization focused on the totally shared variance and proposed the General Factor of Personality (GFP) [4]. However, the substance of these higher-order factors, especially with regard to the GFP, is discussed in the literature (e.g., [8,9,10,11]). While some authors conceive of this shared variance as substantial, there are important theoretical (e.g., [11]) and empirical (e.g., [12]) arguments against this substantial interpretation. The gist of these more critical viewpoints could be summarized as perceiving of the GFP as impression management or social desirability, which might also be substantial but clearly with a different connotation than personality traits [13]. 

While such taxonomies represent a reduction of a complex reality, psychological measurement operationalizing the taxonomies can be considered as the next step of simplification. This is especially relevant when researchers have to deal with pragmatic restrictions in study designs (e.g., assessment-time), which often lead to time-efficient but narrow operationalisations of psychological constructs. For instance, measurements of reasoning are commonly used to operationalize general intelligence (*g*) [14], or specific tests such as Raven’s Matrices test [15] are applied as operationalisations of reasoning or even *g* [16]. In a similar manner, comprehensive FFM questionnaires such as the NEO-PI-R [17], which allow investigating dimensions and facets, are substituted by shorter versions such as the NEO-FFI [17] or even ultra-short versions such as BFI-10 [18], which do not allow to score the whole breadth of the dimensions with regard to the underlying facets. A consequence of such narrow operationalisations leads to the fact that abilities and personality traits are not assessed in their full broadness, with the possibility that only subconstructs at a lower hierarchy level are measured (e.g., figural reasoning as a measurement of *g*).

Much criticism has been expressed regarding too simplistic conceptualizations and measurements of psychological constructs (e.g., [19,20,21]). As emphasized by Ackermann [22], the hierarchical structure of the constructs under investigation is especially important when examining personality-intelligence relations. That means one should not expect the same correlation between personality (e.g., Openness) and a broad operationalization of intelligence (e.g., *g* at a higher hierarchical level) compared to a specific operationalization of intelligence (e.g., figural reasoning at a lower hierarchical level). On the other hand, operationalisations with limited coverage of the personality constructs (e.g., only selected facets of Conscientiousness) will most likely lead to different correlations with intelligence compared to comprehensive personality operationalisations aiming to cover the whole breadth of the construct. These differences related to the hierarchical structure of the constructs can be explained using Wittmann’s [23] Brunswik symmetry principle.

### 1.1. The Brunswik Symmetry Principle

**Figure 1:**
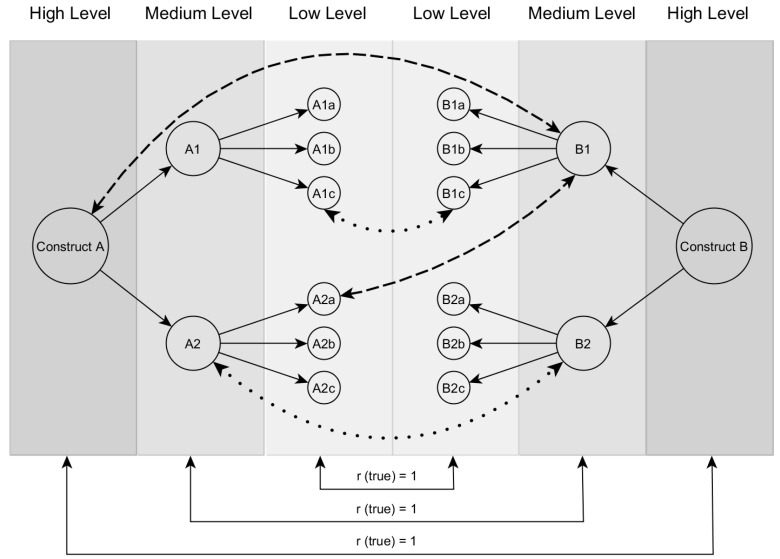
Illustration of the Brunswik symmetry principle according to Wittmann [23,24]. Dotted lines = symmetrical comparison. Dashed lines = asymmetrical comparison.

The Brunswik symmetry principle [23,24] is an adaption of Brunswik’s lens model [25] to describe the relations between hierarchical constructs at different levels of aggregation (or generalization). According to this principle, it is assumed that the empirical correlation between scores for two constructs underestimates the true correlation if the scores’ hierarchical levels within the respective, hierarchically organized constructs do not match [26]. The idea of the Brunswik symmetry principle is illustrated in Figure 1. Let us assume that two hierarchical constructs are perfectly correlated. Accordingly, the observed empirical correlation will also be perfect if the operationalisations are at the same hierarchical level (dotted lines in Figure 1). In this case, a symmetrical comparison of the two constructs is conducted. However, if researchers use operationalisations at different hierarchical levels, an asymmetrical comparison is carried out (dashed lines in Figure 1). In this case, the empirical correlation will be attenuated.

**Figure 2:**
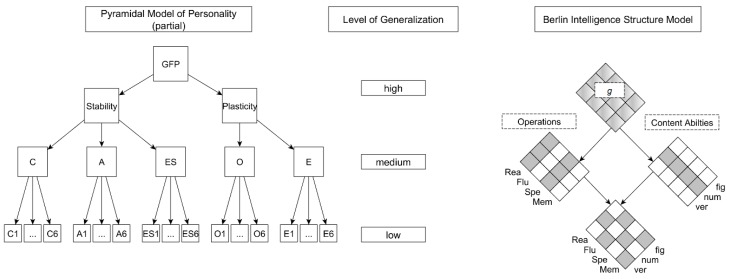
Illustration of the hierarchical levels. GFP = general factor of personality, C = Conscientiousness, A = Agreeableness, ES = Emotional Stability, O = Openness, E = Extraversion, Numbers = facets of the corresponding dimensions, Rea = reasoning, Flu = fluency, Spe = perceptual speed, Mem = short-term memory, ver = verbal, num = numerical, fig = figural. The lowest level (i.e., items and specific responses) of the Pyramidal Model of Personality [4,5] are not displayed.

There are two reasons for this effect. First, a lower (higher) aggregation level narrows (broadens) the breadth of the constructs. As the contents of the construct are different depending on the hierarchical level, the overlap of the constructs is also different. For example, the FFM dimension Openness (medium level, see Figure 2) is compared to the *g*-factor of intelligence (high level, see Figure 2) based on numerical, figural and verbal intelligence (medium level, see Figure 2). In the study of Ashton et al. [27], verbal intelligence was strongly associated with Openness (*r* = 0.37) whereas numerical intelligence was not related to Openness (*r* = 0.08; for possible theoretical explanations of the differences, see [27]). However, a *g*-factor based on numerical, figural and verbal intelligence showed a lower association with Openness (*r* = 0.29) than verbal intelligence alone. From the perspective of the Brunswik symmetry principle [23], these results can be explained by the *g*-factor variance related to numerical intelligence, which was not correlated with Openness. In this example, the *g*-factor at a higher hierarchical level therefore had a smaller content overlap with Openness than verbal intelligence on a lower hierarchical level. Of course, there are other examples in which the *g*-factor might show a larger content overlap and therefore empirical correlation, than more specific abilities on a lower level. However, it should be emphasized that it is often difficult and not straightforward to determine ad hoc a symmetrical comparison of the constructs [28]. 

The second reason is based on the different reliability related to different aggregation levels. In general, higher levels are associated with higher reliabilities compared to lower levels: This might be due to a larger number of items in high-level constructs, suppressed unwanted systematic variance, or averaged error variance [23]. As reliability sets the boundaries to validity, lower hierarchical constructs show generally lower correlations than higher hierarchical constructs (given differing reliabilities).

The usefulness of the Brunswik symmetry principle has been demonstrated in several studies in different research areas, for example by looking at the relations between different cognitive abilities (e.g., [24,29,30,31]), predicting academic and scholastic performance with intelligence or personality (e.g., [32,33,34,35,36]) and predicting occupational and other behavioural criteria with personality (e.g., [37,38,39])^2^. However, as Ackermann [22] highlighted, the Brunswik symmetry principle has been hardly considered with regard to personality-intelligence relations. As a consequence, it is yet unclear how a symmetrical comparison looks like for personality-intelligence correlations. Is the general factor of personality (GFP) symmetrical to the *g*-factor of intelligence, or should we expect the highest correlation between FFM dimensions and *g*? Looking from the other side, are specific abilities such as reasoning or verbal intelligence more symmetrical to the FFM dimensions or to the more specific FFM facets? We do not know yet.

### 1.2. Empirical Studies on Personality-Intelligence Relations

There is a large literature investigating personality-intelligence relations (for comprehensive reviews, see for example [41,42,43]). As Ackermann [22] summarized, correlations between personality and intelligence rarely exceed *r* = 0.20, whereas the strongest personality-intelligence relation can be expected with regard to Openness. However, considering the Brunswik symmetry principle might provide a more fine-grained picture for the association between personality and intelligence.

As outlined above, it is quite common to use narrow operationalisations of personality traits or abilities. Therefore, systematic comparisons of personality and intelligence constructs at different levels are sparse. Nevertheless, there are a few findings which support the potential utility of the Brunswik symmetry principle with regard to personality-intelligence relations. For example in Ackerman and Heggestad’s [41] meta-analysis, general intelligence was substantially related to Openness (*r* = 0.33) but not to Extraversion, Agreeableness, or Conscientiousness (all *r* < 0.10). However, a few additional substantial associations were found at the level of more specific abilities. For example, crystallized intelligence (*r* = 0.11) and fluency (*r* = 0.14) were related to Extraversion, and numerical intelligence (*r* = −0.15) was correlated with Conscientiousness. On the other hand, none of the specific abilities had a higher correlation with Openness than general intelligence (*r* = 0.33). In detail, reasoning (*r* = 0.08), mental speed (*r* = −0.05) and numerical intelligence (*r* = 0.01) showed no correlation, whereas crystallized intelligence (*r* = 0.30) and visual perception (*r* = 0.24) were substantially related to Openness. 

More detailed insights in the relation between Openness and reasoning were found in the study of Beauducel et al. [14]. In detail, a broad operationalization of reasoning showed a substantial correlation with Openness (*r* = 0.23) whereas a narrow operationalization (i.e., figural reasoning) showed no significant correlation. Furthermore, Moutafi, Furnham and Crump [44] investigated the relation between the dimension and facets of Openness and reasoning. Whereas the dimension showed a weak correlation (*r* = 0.09), the facet Openness for new Ideas was substantially related (*r* = 0.20). However, the other facets of Openness showed no or only weak correlations (*r*s < 0.10). In a recent study, Rammstedt, Lechner and Danner [45] reported that FFM facets explained more variance in figural reasoning and verbal knowledge than the FFM dimensions. Similar to previous studies, different facets of the same dimension showed very different relations with intelligence pointing to heterogeneous personality-intelligence correlations on the facet level. 

In summary, it seems worthwhile to consider the Brunswik symmetry principle when investigating the relation between personality and intelligence. Although there are further examples in the literature which provide fragmental evidence (e.g., [27,46,47,48,49,50,51]), there is no systematic examination of the utility of the Brunswik symmetry principle with regard to personality-intelligence relations, yet. It has to be stressed here that the Brunswik symmetry principle does not explain the existence of personality-intelligence correlations but rather helps to optimize the empirical foundations for exploring such estimates.

### 1.3. The Present Study

The primary aim of the present study is to examine the utility of the Brunswik symmetry principle [23] for examining the relations between intelligence and personality. Based on previous evidence regarding the usefulness of the Brunswik symmetry principle in other research areas, we expected that some combinations of specific aggregation levels lead to different correlations than other combinations. Despite the above-mentioned criticism with regard to higher-order factors of personality, we investigated a broad range of construct levels in order to exemplify the Brunswik symmetry principle. In addition, we wanted to examine whether the often reported small or even non-existent relations are potential underestimations caused by asymmetrical operationalisations. In particular, we wanted to test whether we find stronger correlations for some combinations than usually reported in the literature. 

It should be noted that there has been no systematic investigation of the Brunswik symmetry principle with regard to personality-intelligence relations. In combination with a lack of theoretical expectations about most of the combinations under investigation (for some exceptions, see e.g., References [42,52,53,54]), we thus consider the present study as exploratory. In fact, it is not even clear whether the hierarchical structure of intelligence and personality are congruent (i.e., whether the highest level of intelligence corresponds to the highest level of personality, see Figure 2). Therefore, our aim is not to test specific hypothesis but rather to raise awareness of this problem per se and thereby stimulate hypotheses for future research [55].

In order to do so, we used the integrative data analysis (IDA) approach [56] and combined several data sets used in previously published studies [52,57,58,59,60]. In the first study, we used a comprehensive measurement of personality, differentiating between the most widely used hierarchical levels of personality (i.e., GFP, Big Two, FFM dimensions, facets) as well as a commonly used measurement of intelligence differentiating two lower levels (i.e., general reasoning at a medium level and content-specific reasoning at a low level; see Figure 2). In the second study, we used a short version of the personality questionnaire (i.e., discriminating between GFP and the Big Two at a high level and FFM dimensions at a medium level) in combination with a comprehensive measurement of intelligence providing information at three different levels (see Figure 2). The research question we are investigating in this paper has not been addressed in any of the original publications.

## 2. Study 1

In the first study, we combined data sets from three different studies [52,58,60] featuring a comprehensive personality measurement and a commonly used but less discriminating assessment of reasoning. Therefore, Study 1 particularly focused on a fine-grained differentiation of personality (i.e., facets, dimension, Big Two, GFP; see Figure 2) to examine the utility of the Brunswik symmetry principle.

### 2.1. Materials and Methods 

#### 2.1.1. Participants

The total sample size was *N* = 694 (*N*_1_ = 243^3^, *N*_2_ = 180, *N*_3_ = 271). Participants were high school students (German Gymnasium; [58]) or psychology students enrolled at a German university [52,60]. Twelve participants were excluded from the analyses because of failed validity checks of their NEO-PI-R responses according to the test manual (8) or because of completely missing values on intelligence (3) or gender (1), Thus, the final sample size was *N* = 682. The average age was 20.5 years (*SD* = 5.31, *Min* = 15, *Max* = 45) and 66.9% of the participants were female.

#### 2.1.2. Materials

Personality was assessed with the 240 items of the German version of the NEO-PI-R [62], which differentiates between the five dimensions (Openness, Conscientiousness, Extraversion, Agreeableness, Neuroticism) and six facets for each dimension. In addition, higher-order factors (i.e., Big Two and GFP) can be calculated. The NEO-PI-R is probably one of the most used questionnaire in personality research. Reliability and construct validity of the NEO-PI-R can be considered as good [62]. However, the psychometric quality in terms of factorial validity is unsatisfying (e.g., [63,64]) and, as outlined below, the findings of the present study did not provide counterevidence.

Intelligence was assessed with the basic module of the Intelligence Structure Test 2000 R (IST-2000-R) [65]. In detail, three time-limited subtests for each content domain (i.e., verbal, figural, numerical) of reasoning were applied. In total, 180 items (20 items per subtest) were administered. Besides general reasoning, three more specific abilities were considered: verbal reasoning, figural reasoning and numerical reasoning.

In each original study, further measurements were applied that are not of interest for the present research question. For more details and information about the study procedure, please consider the publications of the original studies [52,58,60].

#### 2.1.3. Statistical Analysis

We combined the three different data sets to one data set, which was used for our analysis. This integrative data analysis (IDA) approach [56] has several advantages compared to meta-analysis based on summary statistics. For example, combining different data sets leads to increased sample heterogeneity and increased statistical power, which is especially important in research areas with small effects sizes such as personality research [66]. In addition, it was particularly important for our research question to examine various levels of the hierarchical constructs by aggregating the individual data in different ways. However, it should be noted that the present study is not a meta-analysis based on individual data in a strict sense as described by Cooper and Patall [67]. Although we comprehensively searched in the literature to identify relevant studies, our aim was not to include all possible studies. Instead, we focused on recent data sets including the same measurements (i.e., NEO-PI-R and IST-2000-R), for which a hierarchical structure has been established.

Based on the full data set, we used the 240 items of the NEO-PI-R to calculate a mean score for each facet (i.e., 30 facets, low level), which were used to calculate mean scores for the broader five dimensions (medium level). We reversed Neuroticism into Emotional Stability for our analysis in order to facilitate the interpretation of the findings (i.e., the same direction of correlations across the hierarchical levels of personality). Based on the dimensions, the higher-order factors Stability (Emotional Stability, Agreeableness and Conscientiousness) and Plasticity (Extraversion and Openness) were calculated as mean scores for the Big Two, which were then used to calculate a mean score for the GFP (high level). With regard to the IST-2000-R, we used aggregated scores for verbal reasoning, figural reasoning and numerical reasoning (low level) each based on three subtest scores. We calculated the score for general reasoning (medium level) as an average of the three lower level scores. In the next step, bootstrapped (number of draws = 1000) Pearson correlations between the scores at various aggregations levels and bootstrapped 95% confidence intervals (CI) were calculated. As we consider the present study as exploratory [55], we do not present *p*-values but rather interpret CIs as plausible values of personality-intelligence correlations in the population [68]. In detail, we focus on effect sizes that are interpreted as small (|*r*| ≥ 0.10), medium (|*r*| ≥ 0.20) and large (|*r*| ≥ 0.30) according to Gignac and Szodorai [66]. 

In order to evaluate the usefulness of the Brunswik-Symmetry, we evaluated the differences between the correlations associated with the different aggregation levels. For example, to investigate whether the correlation between personality and general reasoning was different compared to the correlation between personality and content-specific reasoning (e.g., verbal reasoning), the differences between the correlations were calculated. Following Cohen’s [69] effect size guidelines regarding the differences between correlations, we considered a difference of |*r*_diff_| ≥ 0.10 as substantial^4^. The differences scores were calculated based on Wilcox’ [70] bootstrap approach of dependent and overlapping correlations (number of draws = 1000)^5^. Following an equivalence testing approach [71], we deemed a difference as substantial if the 90% confidence interval included the critical effect size of |*r*_diff_| = 0.10 and the 95% confidence interval did not include zero [72].

Gender was controlled for in all analyses as gender differences were reported both for personality (e.g., [73]) and intelligence (e.g., [74,75]). In general, the results were similar (personality-intelligence relations) or slightly stronger (evaluation of the Brunswik symmetry) if gender was not controlled for.

It should be noted that we did not report the results of latent analysis for several reasons. First, although the final sample size was relatively large compared to previous studies (for an overview, see e.g., Reference [41]) and sufficient in order to achieve stable correlations between personality and intelligence [76], it was still too small to achieve stable latent estimations for the personality measurement. As demonstrated by Hirschfeld, Brachel and Thielsch [77], even sample sizes exceeding 1000 participants do not result in stable factor loadings for less comprehensive FFM questionnaires than the NEO-PI-R. Second, the psychometric validity of the NEO-PI-R can be considered as insufficient (e.g., [63,64]). In line with previous studies, several strategies to conduct latent analysis (e.g., separate measurement models for each facet or dimension) were not successful for the present study as the model fits were still insufficient. As a consequence, reliable evidence regarding measurement invariance across the original studies [56] was not available. However, in order to control for measurement error and, thus, to get a less biased estimation of the true personality-intelligence relations, we additionally investigated disattenuated correlations [78] based on a bootstrap approach [79]. The differences between uncorrected and corrected correlations were negligible in particular with regard to the evaluation of the Brunswik symmetry principle. The findings related to the disattenuated correlations are presented in the Appendix A.

All analyses were conducted with the R software [80] and in particular with the packages apaTables [81], foreach [82], doParallel [83], ggplot2 [84], gridExtra [85], lavaan [86], psych [87], WRS [88] and xtable [89].

### 2.2. Results

Table 1 displays the descriptive statistics and reliability estimates. The correlations between scores for the different hierarchical levels of personality (i.e., GFP, Big Two, FFM dimensions, facets) and reasoning (i.e., general reasoning and content-specific reasoning) are presented in Table 2 (disattenuated correlations corrected for reliability are shown in Table A1 in the Appendix A).

#### 2.2.1. General Factor of Personality and the Big Two: Stability and Plasticity (High Level)

**Figure 3:**
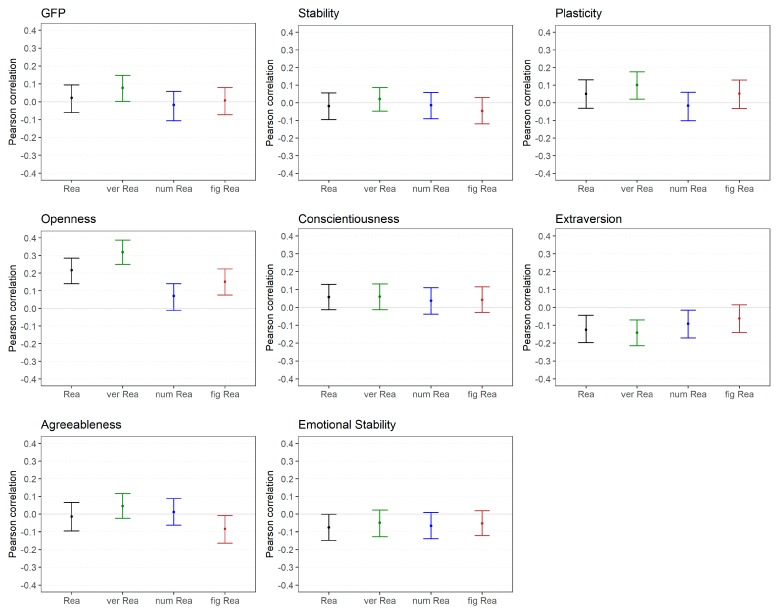
Study 1: Correlations between reasoning and higher-order factor scores as well as FFM dimensions scores of personality. Rea = reasoning, ver = verbal, num = numerical, fig = figural.

Figure 3 displays the correlations between scores for the higher-order factors of personality (i.e., GFP and Big Two) and reasoning scores as well as the five FFM dimension and reasoning scores. With regard to the GFP, none of the reasoning abilities (medium and low level) showed a substantial correlation (Figure 3, upper part).

Stability was not related to reasoning ability (medium and low level). However, Plasticity was substantially associated with verbal reasoning (low level) but not with general reasoning (medium level), numerical reasoning, or figural reasoning (both medium Level; Figure 3, upper part).

#### 2.2.2. FFM Dimensions (Medium Level)

With regard to scores for the FFM dimensions (Figure 3, middle and lower part), no substantial correlations were observed between Conscientiousness, Agreeableness and Emotional Stability and reasoning scores (medium und low level). Extraversion showed a small negative correlation with general reasoning (medium level) and verbal reasoning (low level) but no substantial correlation with numerical reasoning or figural reasoning (both low level). A more differentiate pattern was observed with regard to Openness. Verbal reasoning (low level) was largely, general reasoning (medium level) moderately, figural reasoning (low level) weakly and numerical reasoning (low level) not substantially correlated with the Openness dimension, respectively. In summary, only Openness and Extraversion showed substantial relations with intelligence, which was most evident for verbal reasoning.

#### 2.2.3. FFM Facets (Low Level)

**Figure 4:**
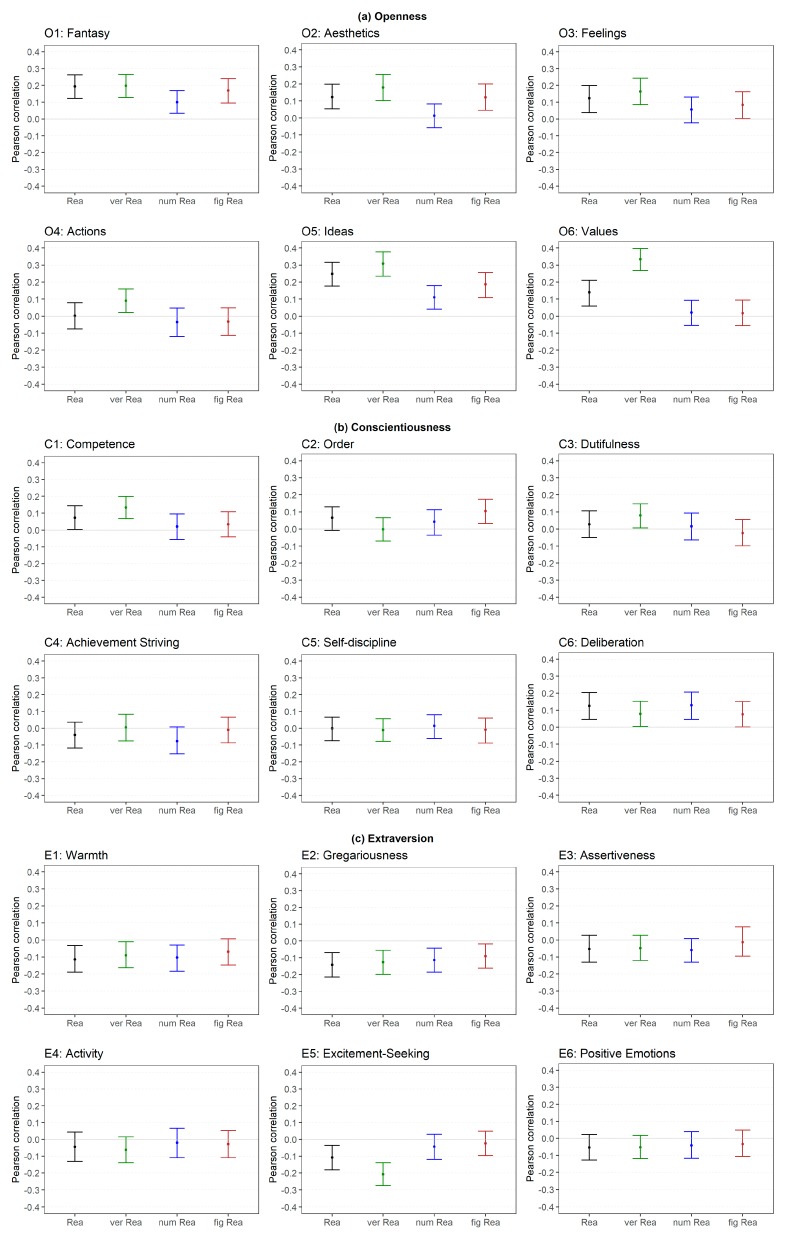
Study 1: Correlations between reasoning and the facets of (**a**) Openness, (**b**) Conscientiousness and (**c**) Extraversion. Rea = reasoning, ver = verbal, num = numerical, fig = figural.

**Figure 5:**
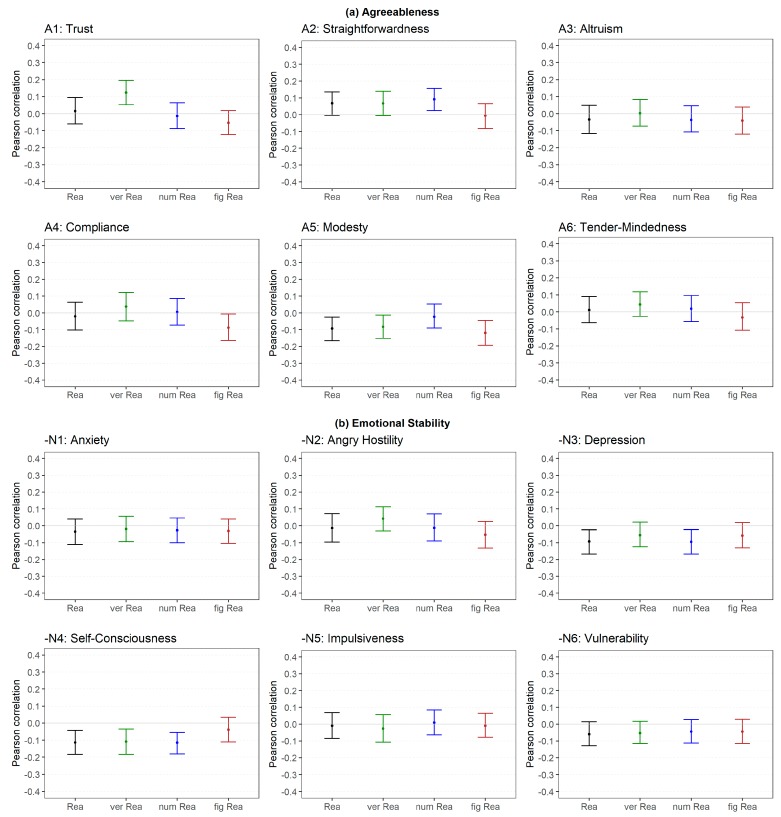
Study 1: Correlations between reasoning and the facets of (**a**) Agreeableness and (**b**) Emotional Stability. Rea = reasoning, ver = verbal, num = numerical, fig = figural.

The correlation between the facets and reasoning are presented in Figure 4 and Figure 5. With regard to Openness (Figure 4a), the facet Fantasy showed a moderate correlation with verbal reasoning (low level) and a small correlation with general reasoning (medium level) as well as with numerical and figural reasoning (both low level). Aesthetics was weakly correlated with all abilities except for numerical reasoning (low level). Feelings showed a small correlation with general reasoning (medium level) and verbal reasoning (low level) but no correlation with the other low-level abilities. Actions were not substantially correlated with reasoning (medium and low level). Ideas was largely correlated with verbal reasoning (low level), moderately related to general reasoning (medium level) and weakly correlated to numerical and figural reasoning (both low level). Values showed a large relation to verbal reasoning (low level) and a small correlation with general reasoning (medium level) but no relation with numerical or figural reasoning (both low level). In summary, the facets Ideas and Values showed the highest correlations across all abilities and verbal reasoning showed the highest correlation across all facets.

The facets of Conscientiousness (Figure 4b) where not substantially correlated with reasoning except for a few rather unsystematic small correlations (e.g., Deliberation). 

With regard to Extraversion (Figure 4c), the direction of all correlations was negative. Warmth was weakly related with general (medium level) and numerical reasoning (low level). Gregariousness showed a small correlation with all abilities except for figural reasoning (low level). Assertiveness, Activity and Positive Emotions were not related to reasoning at all. Excitement-Seeking showed a medium correlation with verbal reasoning (low level) and a small correlation with general reasoning (medium level) but not with numerical or figural reasoning (low level). In summary, the facet Gregariousness was associated with a broad range of abilities but the other facets were not systematically related to reasoning. However, the largest (isolated) relation was found for Excitement-Seeking and verbal reasoning.

With regard to the facets of Agreeableness (Figure 5a), there were no substantial relations with reasoning except for a few isolated small correlations.

The facets of Emotional Stability (Figure 5b) were not related to reasoning except for Self-consciousness (reversed), which showed a small association with all abilities except for figural reasoning (low level). 

#### 2.2.4. The Brunswik Symmetry Principle

**Figure 6:**
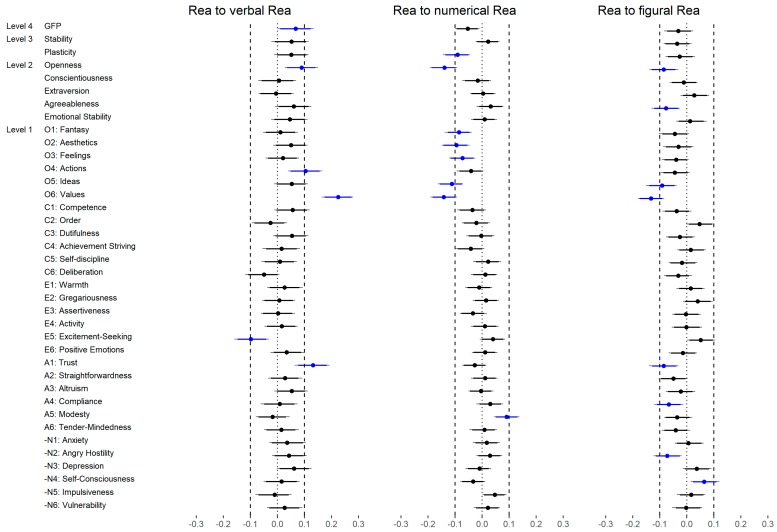
Study 1: Evaluation of the Brunswik Symmetry principle with regard to reasoning. Change of correlations when specific reasoning abilities (low level) were considered instead of general reasoning (medium level). Rea = Reasoning. Blue lines indicate substantially different correlations based on an equivalence testing approach. The facets of Neuroticism (-N) were reversed to be in line with the dimension of Emotional Stability.

To evaluate the utility of the Brunswik Symmetry principle, we separately considered the different hierarchical levels of personality and reasoning. With regard to reasoning, we examined whether the correlation between personality and general reasoning (medium level) was different compared to the correlations between personality and specific reasoning abilities (low level). Based on the bootstrapped difference scores of correlations (i.e., general reasoning—verbal reasoning; general reasoning—numerical reasoning; general reasoning—figural reasoning) across all possible levels of personality (i.e., GFP, Big Two, FFM dimensions, facets) we evaluated the change of correlations. Figure 6 provided a summary of the results. 

In total, we found 22 out of 114 (19%) correlations which were substantially different if the specific reasoning abilities (low level) were considered instead of general reasoning (medium level). Most of these differences were negative (68%), meaning that the specific reasoning abilities showed a weaker relation with personality than general reasoning. In detail, general reasoning and verbal reasoning showed more or less the same association with personality: Only 6 out of 38 (16%) correlations were substantially different if verbal reasoning was used instead of general reasoning. These differences were mostly positive indicating that in these cases verbal reasoning showed a higher correlation with personality than general reasoning. With regard to numerical reasoning and figural reasoning, 8 (21%) substantial differences were found in each of them. Please note that these differences were systematically negative and, in the case of numerical reasoning, related to Openness (i.e., numerical reasoning was less related to Openness than general reasoning).

**Figure 7:**
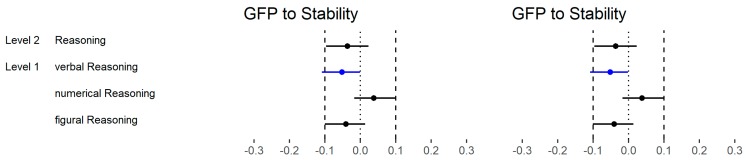
Study 1: Evaluation of the Brunswik Symmetry principle with regard to personality. Change of correlations when Stability and Plasticity (Big Two) were considered instead of GFP (both high level). Blue lines indicate substantially different correlations based on an equivalence testing approach.

With regard to the higher-order factors of personality and across all abilities (Figure 7), 1 out of 8 (13%) correlations were substantially different if Stability and Plasticity (Big Two) were considered instead of the GFP (both high level). However, the difference only just exceeded our criteria of substantial differences.

**Figure 8:**
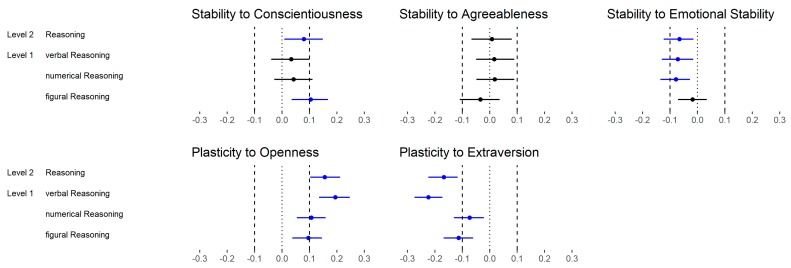
Study 1: Evaluation of the Brunswik Symmetry principle with regard to personality. Change of correlations when FFM dimensions (medium level) were considered instead of Stability and Plasticity (Big Two; high level). Blue lines indicate substantially different correlations based on an equivalence testing approach.

With regard to the FFM dimensions (Figure 8), 13 out of 20 (65%) correlations were substantially different if the dimensions (medium level) were considered instead of the Big Two (high level). However, whereas 5 out of 12 (42%) correlations were different with regard to Stability, all correlations were substantially different regarding Plasticity. That means that Openness and Extraversion showed systematically stronger relations with reasoning than Plasticity (i.e., from mostly zero correlations to small negative correlations for Extraversion). This also means that the relation between Plasticity and reasoning was mainly due to Openness variance.

**Figure 9:**
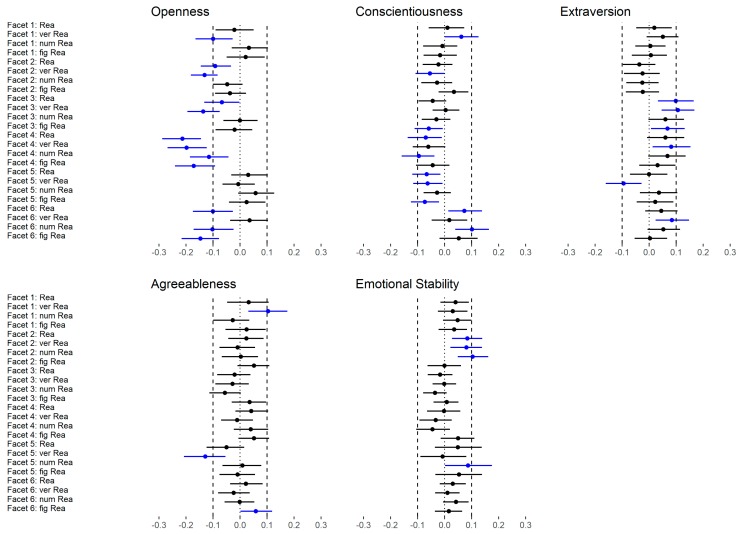
Study 1: Evaluation of the Brunswik Symmetry principle with regard to personality. Change of correlations when facets (low level) were considered instead of FFM dimensions (medium level). Rea = reasoning, ver = verbal, num = numerical, fig = figural. Blue lines indicate substantially different correlations based on an equivalence testing approach.

In the last step, we examined the correlations between the FFM dimensions (medium level) and reasoning as well as the correlations between the FFM facets (low level) and reasoning (Figure 9). In total, 35 out of 120 (29%) were substantially different, whereas most of the effects were negative (83%; please note that Extraversion was negatively associated with reasoning, see Table 2). With regard to Openness, 12 out of 24 (50%) correlations were substantially different if the facets were considered instead of the dimension. All differences were negative meaning that the facets showed systematically lower relations with reasoning than the corresponding dimension. A similar pattern was found for Conscientiousness (10 out of 24; 42%) and Extraversion (6 out of 24; 25%). With regard to Conscientiousness, most of the differences were caused by changes from a non-substantial positive relation to a non-substantial negative relation (see Table 2). With regard to Extraversion, the facets showed mostly no substantial correlation whereas the dimension showed a small negative correlation (see Table 2). The differences between the dimension and facets of Agreeableness and Emotional Stability were rather negligible (3 out of 24 (13%) and 4 out of 24 (17%), respectively).

##### 2.3. Summary

The aim of the first study was to examine the utility of the Brunswik symmetry principle [23] specifically with regard to a fine-grained differentiation of personality (i.e., facets, FFM dimensions, Big Two, GFP). The findings provided evidence that considering the Brunswik symmetry principle allows detailed insights into personality-intelligence relations. Overall, general reasoning (medium level) showed a higher correlation with personality than content-specific reasoning (low level). It is worth noting that numerical reasoning showed a systematically lower correlation with Openness than general reasoning. Furthermore, considering figural reasoning instead of general reasoning substantially decreased the personality-intelligence relation in 21% of the combinations. 

With regard to the hierarchical structure of personality, systematic and substantial correlations were only found for Openness and Extraversion, while the dimensions (medium level) showed larger associations than the facets (low level), the Big Two and GFP (high level). However, substantial correlations were also found for some facets (low level) with regard to the other FFM dimensions. In general, the highest correlations between Openness and reasoning (*r*_max_ = 0.33, corrected for unreliability 0.52, see Table A1) and Extraversion and reasoning (*r*_max_ = −0.21, corrected for unreliability −0.30, see Table A1) were larger in our study compared to the meta-analytically derived correlation (*r* = 0.08 and *r* = 0.06 for Openness and Extraversion, respectively [41]), indicating that specific combinations of hierarchical levels can lead to substantial personality-intelligence relations. 

## 3. Study 2

In the second study, we combined data sets from two different studies [57,59] featuring an elaborated measurement of intelligence and a shorter version of the personality questionnaire. Therefore, Study 2 focused on a fine-grained differentiation of intelligence in order to examine the utility of the Brunswik symmetry principle (see Figure 2). Noteworthy, some of the combinations of aggregation levels were equivalent to Study 1 (i.e., GFP, Big Two as well as FFM dimensions of personality and general as well as content-specific reasoning). Therefore, these conditions can be considered as a replication of Study 1 with the main difference being that the personality measures no longer cover all of the content covered in Study 1.

### 3.1. Method

#### 3.1.1. Participants

The sample size was *N* = 414 (*N*_1_ = 122, *N*_2_ = 292). Participants were high school students (German Gymnasium; [59]) or part of a convenience sample with different educational and occupational backgrounds [57]. One participant was excluded from the analyses because of completely missing values on personality. Thus, the final sample size was *N* = 413. The average age was 21.9 years (*SD* = 12.1, *Min* = 14, *Max* = 61) and 56% of the participants were female.

#### 3.1.2. Materials

The German NEO-FFI [90], a short version of the NEO-PI-R with 60 items, was applied. In contrast to the NEO-PI-R, the NEO-FFI differentiates between the five broad dimensions without considering the facets. In addition, higher-order factors (Big Two, GFP) can be calculated by aggregating the scores of the five dimensions. Research on the NEO-FFI showed satisfying evidence regarding reliability and construct validity [90]. However, the psychometric issues associated with NEO-PI-R (see above) also have been found for the NEO-FFI (e.g., [91]).

With regard to intelligence, the Berlin Intelligence Structure (BIS) test ([92]; for an English description, see [2]) was applied. The BIS test is a comprehensive operationalization of the BIS model [1,93] (see Figure 2) with 45 time-limited tests. Based on the test scores, we calculated aggregated scores according to the test manual. In detail, we used the *g*-factor (high level), four scores for cognitive operations (i.e., reasoning, fluency, perceptual speed, short-term memory) and three scores for content-based abilities (i.e., verbal intelligence, numerical intelligence, figural intelligence) (medium level) and 12 scores each as a combination of operations and content (e.g., verbal reasoning, figural short-term memory) as low-level abilities^6^.

For more details and information about the study procedure, please consider the publications of the original studies [57,59].

#### 3.1.3. Statistical Analysis

The statistical approach was the same as described in Study 1. Both data sets were combined into a large data set for further analyses. The 60 Items of the NEO-FFI were used to calculate the five dimensions (i.e., Neuroticism was reversed into Emotional Stability). Based on the dimensions, the higher-order factors Stability and Plasticity were calculated as mean scores for the Big Two, which were then aggregated to the mean score for the GFP. With regard to the BIS test, we used the 45 test scores to calculate the various aggregated scores according to the test manual [92]^7^. 

### 3.2. Results

Descriptive statistics and reliabilities are displayed in Table 3. The correlations between personality (i.e., GFP, Big Two and FFM dimensions) and the abilities at different levels of aggregation are presented in Table 4 (disattenuated correlations corrected for reliability are shown in Table A2 in the Appendix A). 

#### 3.2.1. General Factor of Personality and the Big Two: Stability and Plasticity (High Level)

**Figure 10:**
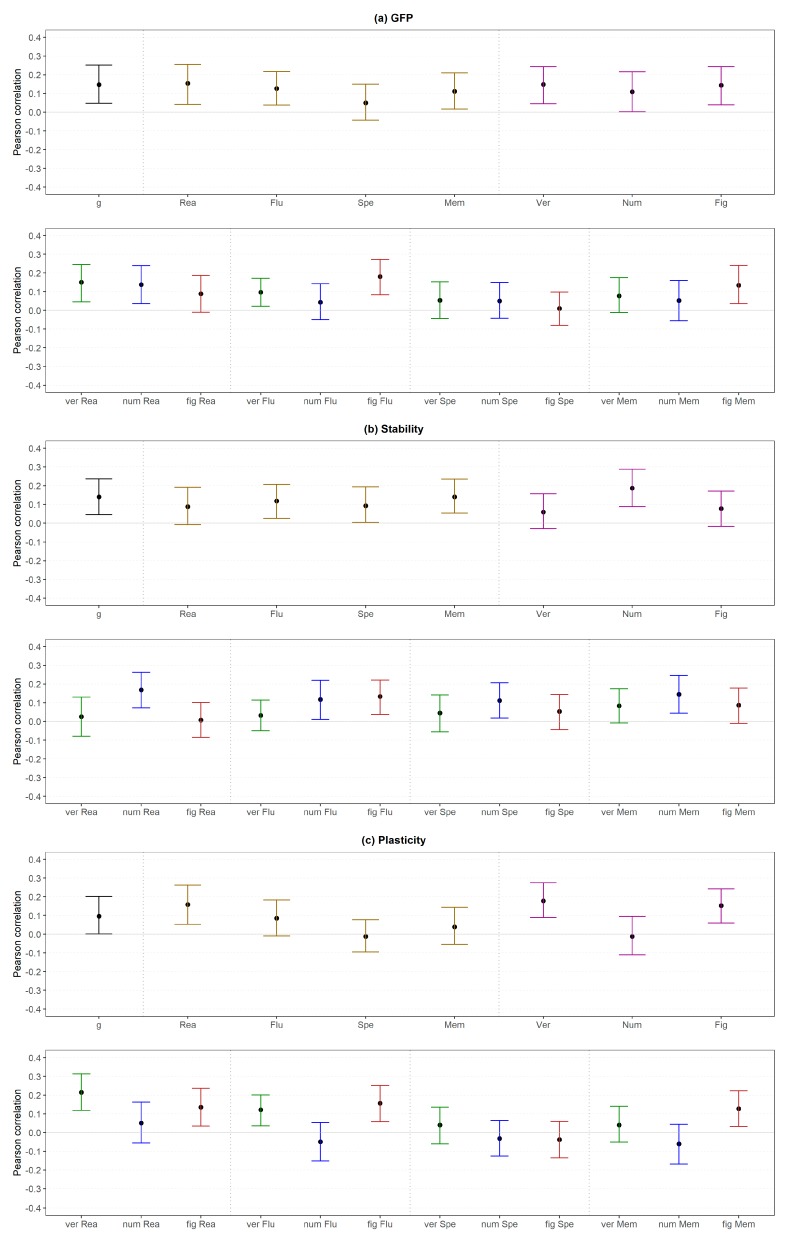
Study 2: Correlations between intelligence and higher-order factors of personality: (**a**) GFP, (**b**) Stability, (**c**) Plasticity. Rea = reasoning, Flu = fluency, Spe = perceptual speed, Mem = memory, Ver = verbal (intelligence), Num = numerical (intelligence), Fig = figural (intelligence).

As displayed in Figure 10a (upper part), all broad abilities (high and medium level) except for perceptual speed (medium level) were substantially related to the GFP. With regard to more specific abilities (low level; Figure 10a, lower part), two of four abilities related to verbal content were weakly associated with GFP (i.e., verbal reasoning and verbal fluency). In addition, numerical reasoning, figural fluency and figural memory showed also a small correlation. The results related to reasoning were only partly in line with those in Study 1 as none of the correlations were substantial in Study 1.

Stability was substantially and weakly related to *g* (high level), fluency, perceptual speed, memory and numerical intelligence (medium level) but not to the other broader abilities (medium level; Figure 10b, upper part). In addition, all numerical abilities and figural fluency (all low level) showed a substantial and weak association with Stability (Figure 10b, lower part).

Plasticity was substantially and weakly correlated with *g* (high level), reasoning, verbal intelligence and figural intelligence (all medium level; Figure 10c, upper part). With regard to the more specific abilities (low level; Figure 10c, lower part), verbal reasoning showed a medium association, whereas three out of four figural abilities and verbal fluency showed a weak association. The other abilities were not substantially related to Plasticity.

Again, the results related to reasoning were only partly in line with those in Study 1. In detail, there was only one out of eight substantial correlations in Study 1 (i.e., verbal reasoning and Plasticity) whereas in Study 2 four out of eight associations were considered as substantial.

#### 3.2.2. FFM Dimensions (Medium Level)

**Figure 11:**
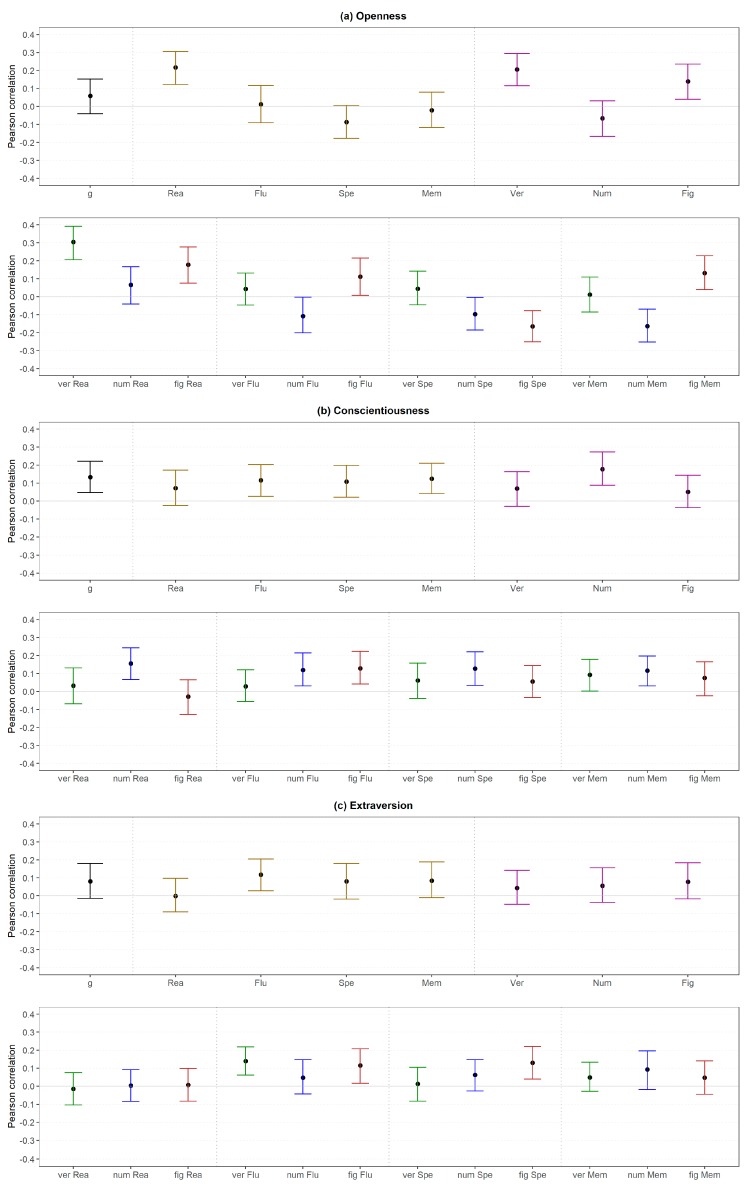
Study 2: Correlations between intelligence and (**a**) Openness, (**b**) Conscientiousness and (**c**) Extraversion. Rea = reasoning, Flu = fluency, Spe = perceptual speed, Mem = memory, Ver = verbal (intelligence), Num = numerical (intelligence), Fig = figural (intelligence).

With regard to Openness, reasoning and verbal intelligence showed a medium and figural intelligence a small association (all medium level), whereas other broader abilities (high and medium level) had no substantial relation to the Openness dimension (Figure 11a, upper part). On the more fine-grained lower level (Figure 11a, lower part), verbal reasoning showed the highest correlation with Openness (large effect size), whereas the other verbal abilities were not substantially related to Openness. All abilities related to figural stimuli showed a small correlation but the directions were different depending on the cognition (i.e., positive for reasoning, fluency and memory; negative for perceptual speed). In addition, except for numerical reasoning, all numerical abilities were weakly and negatively related to Openness. Thus, not only the effect size but also the direction of the effect was dependent on the specific ability. The findings related to reasoning were in line with those in Study 1.

Conscientiousness showed a small correlation with *g* (high level), fluency, perceptual speed, memory and numerical intelligence (all medium level). However, reasoning, verbal intelligence and figural intelligence (all medium level) were not substantially related to this FFM dimension (Figure 11b, upper part). Lower-level abilities showed generally similar associations with Conscientiousness (Figure 11b, lower part). This means that all abilities related to numerical content were weakly related to Conscientiousness. In addition, figural fluency showed a small correlation. In contrast to Study 1, numerical reasoning was substantially related to this FFM dimension; the remaining findings were similar in both studies.

With regard to Extraversion, there was only a small association with fluency (medium level) but no further association with abilities at a high or medium level (Figure 11c, upper part). A closer look at low-level abilities confirmed the previous finding; that means verbal and figural fluency were related to this FFM dimension (Figure 11c, lower part). Additional relations were not found except for figural perceptual speed. This is an interesting finding, as neither figural intelligence nor perceptual speed (both medium level) showed a substantial correlation with Extraversion. The findings were not in line with those in Study 1, as two out of four reasoning abilities were related to Extraversion in the first study but no reasoning ability showed a substantial correlation in the second study.

**Figure 12:**
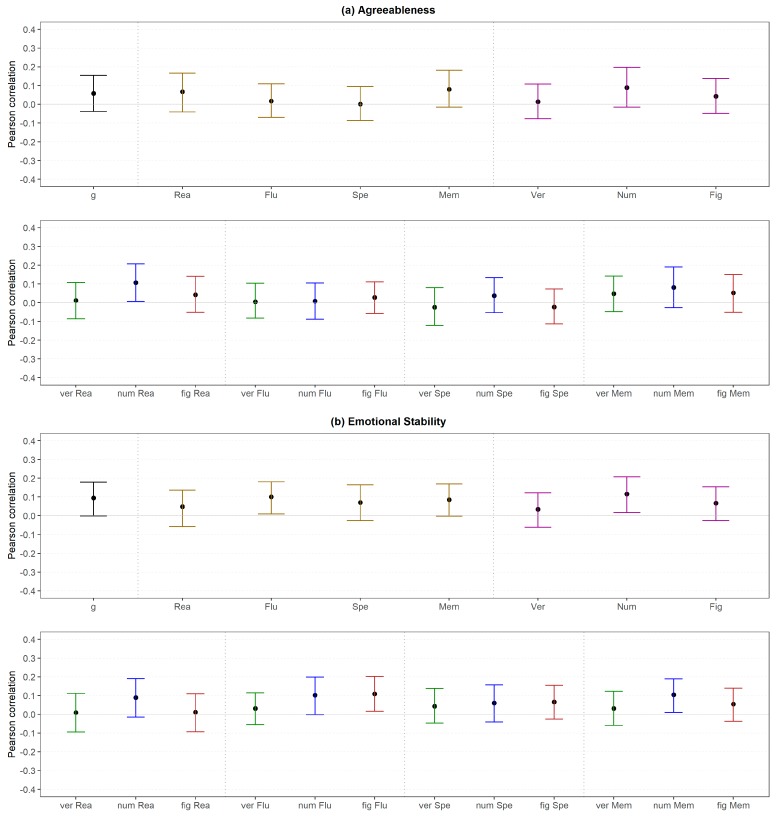
Study 2: Correlations between intelligence and (**a**) Agreeableness and (**b**) Emotional Stability. Rea = reasoning, Flu = fluency, Spe = perceptual speed, Mem = memory, Ver = verbal (intelligence), Num = numerical (intelligence), Fig = figural (intelligence).

Agreeableness was not substantially correlated with any abilities independent of the level (Figure 12a) except for one small association regarding numerical reasoning. Regardless of the latter, these findings were in line with the results in Study 1. 

Only the association between Emotional Stability and fluency as well as numerical intelligence (both medium level) could be considered as substantial (Figure 12b, upper part), although small in terms of our effect size classifications. Correlations between Emotional Stability and low-level abilities were only substantial regarding numerical fluency, figural fluency and numerical memory (Figure 12b, lower part). The findings related to reasoning were in line with those in Study 1.

#### 3.2.3. The Brunswik Symmetry Principle

**Figure 13:**
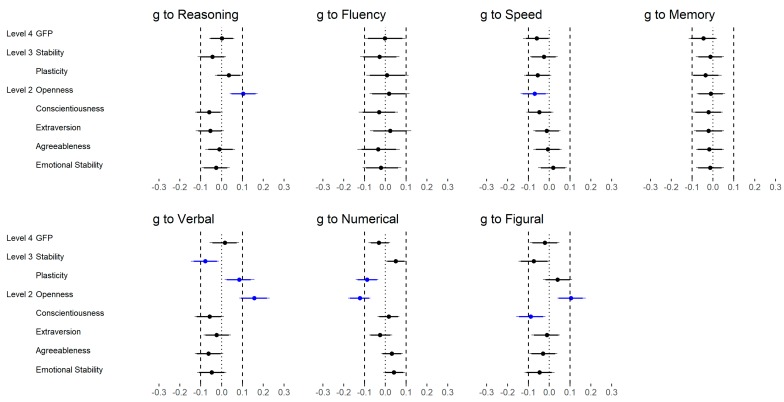
Study 2: Evaluation of the Brunswik Symmetry principle with regard to intelligence. Differences in correlations between the *g*-factor (high level) and specific abilities (medium level). Blue lines indicate substantially different correlations based on an equivalence testing approach.

Similar to Study 1, differences scores of correlations were evaluated. Comparing the correlations between the *g*-factor (high level) and personality with the correlations between medium level abilities and personality led to 9 out of 56 (16%) substantial different associations, which were mixed regarding the direction (i.e., 44% of the differences were negative; Figure 13).

**Figure 14:**
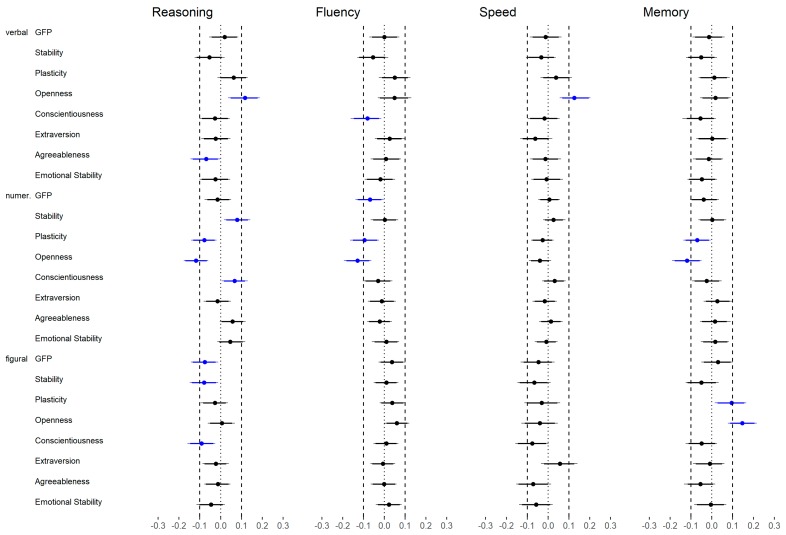
Study 2: Evaluation of the Brunswik Symmetry principle with regard to intelligence. Differences in correlations between specific abilities (medium level) and low-level abilities. Blue lines indicate substantially different correlations based on an equivalence testing approach.

Figure 14 displays the changes when specific abilities (medium level) were compared with the lowest order abilities (low level). In total, only 18 out of 96 (19%) correlations were substantially different. Furthermore, there was a negative tendency with regard to the direction of the effect (i.e., 67% were negative) but no clear pattern regarding specific personality-intelligence combinations. Thus, considering more specific abilities (low level) instead of broader abilities (medium level) did not change the personality-intelligence relations in a systematic manner. 

The findings regarding reasoning (medium and low level) and personality (all levels) were mostly in line with those in Study 1. In detail, verbal reasoning showed a larger relation with Openness than general reasoning in both studies. Furthermore, there were mostly no substantial differences regarding the other combinations of personality and verbal reasoning. However, the findings differed between both studies regarding the GFP (i.e., substantial difference in Study 1, no difference in Study 2) and Agreeableness (i.e., no difference in Study 1, substantial difference in Study 2). With regard to numerical reasoning, the substantial difference regarding Plasticity and Openness and the non-substantial differences regarding GFP, Extraversion, Agreeableness and Emotional Stability were confirmed in Study 2. However, the results differed regarding Stability (i.e., no substantial difference in Study 1, substantial differences in Study 2) and Conscientiousness (i.e., no effect in Study 1; substantial difference in Study 2). An even more mixed pattern was found for figural reasoning (i.e., only the non-substantial differences regarding Plasticity, Extraversion and Emotional Stability were similar in both studies).

**Figure 15:**
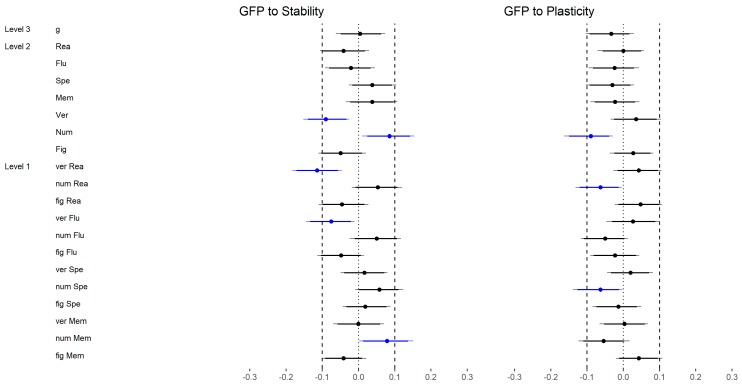
Study 2: Evaluation of the Brunswik Symmetry principle with regard to personality. Change of correlations when Stability and Plasticity (Big Two) were considered instead of GFP (both high level). Blue lines indicate substantially different correlations based on an equivalence testing approach.

With regard to the higher-order factors of personality and across all abilities (Figure 15), 8 out of 40 (20%) correlations were substantially different if Stability and Plasticity instead of the GFP (high level) were considered. Most of the differences were negative (75%).

**Figure 16:**
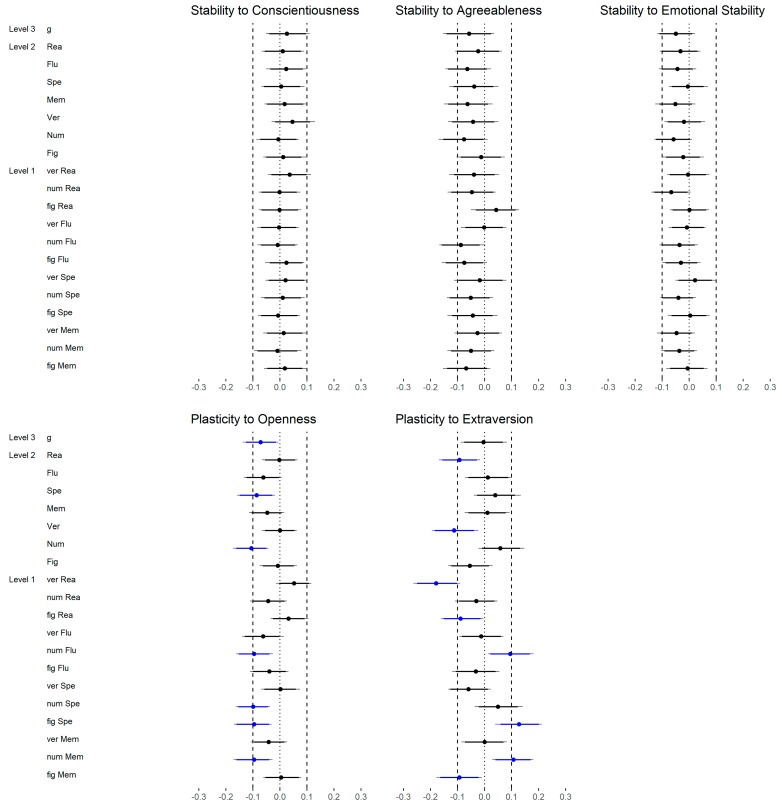
Study 2: Evaluation of the Brunswik Symmetry principle with regard to personality. Change of correlations when FFM dimensions (medium level) were considered instead of the Big Two (high level). Rea = reasoning, Flu = fluency, Spe = perceptual speed, Mem = memory, Ver = verbal (intelligence), Num = numerical (intelligence), Fig = figural (intelligence). Blue lines indicate substantially different correlations based on an equivalence testing approach.

Regarding the FFM dimensions, 15 out of 100 (38%) substantial differences were found if the dimensions (medium level) were considered instead of the Big Two (high level; Figure 16). However, all substantial differences were related to Plasticity and were mostly negative (80%).

### 3.3. Summary

The findings of Study 2 provided further evidence for the utility of the Brunswik symmetry principle with regard to the relation between specific personality and intelligence constructs. On average and in line with Study 1, more general abilities were stronger or at least similarly related to personality than more specific abilities. However, substantial differences were also found between abilities at the same level (e.g., *r* = 0.21 between Openness and reasoning, and *r* = −0.09 between Openness and perceptual speed; see Table 4). Therefore, as long as there is no robust evidence with regard to specific abilities and their relation to personality, it seems to be more reasonable to consider general abilities instead of specific abilities to avoid underestimating the association between personality and intelligence. At the same time and in line with Study 1, correlations regarding specific combinations of low-level abilities with FFM dimensions (medium level) were larger (e.g., *r*_max_ = 0.30 for verbal reasoning and Openness, corrected for unreliability 0.40, see Table A2) compared to the meta-analytically derived correlation (*r* = 0.08 for general reasoning and Openness [41]). Therefore, investigating specific abilities seems to be promising for future research about personality-intelligence relations.

With regard to the hierarchical structure of personality, systematic and substantial associations with intelligence were found for GFP, Stability and Plasticity (both high level), Openness and Conscientiousness (both medium level). Extraversion and Emotional Stability (both medium level) were only infrequently related to specific abilities other than reasoning (e.g., fluency). On average, more general personality traits were stronger or at least similarly related to intelligence than more specific personality traits. However, the highest correlations were found for the Openness dimension (medium level). 

## 4. Discussion

Studies aiming to examine the relation between personality and intelligence often focused on certain personality constructs (e.g., Openness and intelligence vs. Consciousness and intelligence) but did more seldomly consider the hierarchical structure of the constructs (e.g., figural reasoning and personality vs. general reasoning and personality). However, the latter can also substantially influence the correlations between personality and intelligence. According to the Brunswik symmetry principle [23], the highest correlation can be expected if constructs at a similar level are investigated (Figure 1). The present study examined the utility of the Brunswik symmetry principle with regard to personality-intelligence correlations. In doing so, we investigated different combinations of hierarchical levels of personality and intelligence and, thus, exploratively examined which combinations showed the highest association. 

Whereas previous research on the personality-intelligence relations often found no or weak associations (e.g., [97]), our findings demonstrate that correlations between the two construct complexes can be substantially different depending on which specific combinations of hierarchical levels were investigated (e.g., *r* = 0.03 for Openness and *g*, *r* = 0.13 for Openness and reasoning, and *r* = 0.24 for Openness and verbal reasoning). Consequently, some combinations of specific abilities and FFM facets/dimensions were larger than reported in the literature, which did not differentiate on such a fine-grained level (e.g., [41]). Thus, our results provided evidence for the usefulness of the Brunswik symmetry principle [23] for the research of personality-intelligence relations. In the following section, we discuss our findings and provide some general recommendations for future research based on the Brunswik symmetry principle.

### 4.1. Recommendations to Observe Large(r) Personality-Intelligence Relations 

In general, our findings provide further evidence that there is no single personality-intelligence relation hiding somewhere behind a mirage of complexity. Nevertheless, our results suggest that the magnitude of personality-intelligence associations varies across different levels of these hierarchical constructs. This means that researchers should be aware that specific operationalisations of personality and intelligence and thus the choice of specific levels of generalization (e.g., dimensions vs. facets) have a substantial impact on the empirical correlations between personality traits and intelligence. The following recommendations are intended to guide researchers who are interested in maximizing the personality-intelligence correlations.

The relations between higher-order factors of personality and intelligence were mixed in our studies. Only Plasticity and verbal abilities showed a consistent albeit weak correlation in both studies. These mixed results are in line with previous research, which provides evidence against (e.g., [53,54]) or for (e.g., [98,99]) a substantial correlation between higher-order factors of personality and intelligence. However, it should be noted that—apart from other factors discussed in the literature such as differences with regard to personality measurements (e.g., [100]), methods to calculate higher-order factor (e.g., [98]), or subgroups (e.g., [101])—the operationalisations of intelligence regarding an adequate construct representation (e.g., [102]) were quite heterogeneous in previous and our studies making it difficult to compare the results. Thus, the most appropriate conclusion based on our and previous findings would be that more research about the higher-order factors of personality and their relation to intelligence is necessary.

Across both studies and in line with previous research (e.g., [41]), Openness showed the strongest correlation with intelligence. This finding is in line with investment theories (e.g., [52,103,104]), which propose a causal interplay of Openness and intelligence. However, Openness is a heterogeneous construct as the correlations on the facet level differed significantly from no effect to a large effect (see also, e.g., Reference [105]). Therefore, it is generally worthwhile to investigate Openness at a facet level. With regard to intelligence, verbal reasoning on the most specific level is more strongly related to Openness across all other levels of abilities, in general. As a consequence, investigating personality-intelligence relations with the often applied Raven’s Matrices test [15] as a measurement of (figural) reasoning leads to significantly lower correlations. The relations will even be weaker if numerical intelligence tests are used. Future research should also consider that part of the relation between Openness and verbal reasoning that might be attributable to crystallized intelligence. 

Conscientiousness was not related to reasoning in both studies, which is in line with previous meta-analytical research [41] but contrary to some more recent studies showing a small negative correlation (e.g., [44,106]). However, our results suggest positive substantial associations with other cognitive abilities such as fluency, memory, or numerical intelligence, which are less frequently considered regarding the personality-intelligence relations. Thus, focusing on the *g*-factor or reasoning might lead to the premature conclusion that Conscientiousness and intelligence are not related (e.g., no studies investigating Conscientiousness and fluency could be integrated in Ackerman and Heggestad’s meta-analysis [41]). Whether the facets of Conscientiousness provide detailed insights with regard to these cognitive abilities is a question for future research.

The findings with regard to Extraversion and reasoning were not consistent across both studies reflecting the mixed results reported in the literature (e.g., [45,107]). However, in line with previous meta-analytical research [41], we found a substantial relation to fluency and perceptual speed in our second study. Again, we recommend investigating further abilities in addition to the *g*-factor or reasoning. Similar to Conscientiousness, no recommendations with regard to the facet level of Extraversion can be derived from the present study. 

Agreeableness and Neuroticism (Emotional Stability) did not correlate with intelligence in a systematic manner across all hierarchy levels of the constructs. The results regarding Agreeableness were in line with previous meta-analytical results but the findings regarding Neuroticism were not [41]. It has been suggested that the relation between Neuroticism and intelligence arises due to the test-taking situation (i.e., higher test anxiety leads to lower cognitive performance [108]). However, our results based on the FFM facets (Study 1) did not support this assumption, as we found no evidence for a specific correlation between anxiety and cognitive abilities (see also [51]). In summary, the Brunswik symmetry principle seems to be less or not beneficial for these personality-intelligence relations, which can be generally considered as non-existent or weak.

With regard to intelligence, the highest correlations with personality traits were found for the most specific abilities. However, whereas some of these specific abilities showed a strong correlation, other abilities were not related at all. Therefore, a broad assessment of intelligence is generally recommended to investigate personality-intelligence relations. Obviously, possibilities to apply comprehensive and time-consuming measurements such as the BIS test (>2 h) [92] are rather limited. However, considering a facet structure of intelligence as in the BIS model [1,2] with regard to the content abilities (i.e., verbal, numerical, figural) and cognitive operations (e.g., reasoning) enables researchers to use an elaborated but still time-efficient assessment of intelligence. For example, a balanced operationalization as in the first study (i.e., three different subtests per content per cognition) is fairly close to the recommendation regarding a “good *g*” [102] and reduces the risk of unintentionally low correlations. In addition, such an approach allows us to consider different hierarchy levels of intelligence as in the present study. However, if time restrictions are really tight, then we recommend using measurements of verbal intelligence as the chance of increasing the correlation between personality and intelligence is the highest. 

### 4.2. Limitations and Future Directions 

The focus of our study was to examine the utility of the Brunswik symmetry principle [23] with regard to personality-intelligence relations. As the Brunswik symmetry principle seems to be useful at least for some personality traits (i.e., Openness, Conscientiousness, Extraversion) and abilities, there are a couple of issues which we think are particularly important for future studies.

The first issue relates to the operationalization of personality and intelligence. The measurements used in the present study are well established in the scientific community. However, as noted above and in several other studies (e.g., [63,64,91]), there are psychometrical issues regarding the NEO-PI-R and NEO-FFI challenging the application of state-of-the-art statistical approaches such as confirmatory factor analysis [109] in combination with more elaborated measurement models (e.g., [48,110]). Additionally, the current paper also sheds light onto the subtle differences that using different personality measures can cause. Here, we even used only tests from the same test family and still, the findings differed for some relations that could be regarded as replications from Study 1 to Study 2. One reason for this might be the difference in intelligence test used. Yet, such tests tend to have strong convergent validities (e.g., [111,112]). With regard to the NEO-PI-R and NEO-FFI what has to be noted is that the NEO-FFI does not have the same facet balance as the NEO-PI-R. The NEO-PI-R is the result of a long and successful test construction history. In the beginning, efforts were focused on creating items that captured the five domains themselves using complete statements instead of just adjectives. In 1992, Costa and McCrae published the NEO-PI-R, allowing for the assessment of six facets for each of the domains. Thus, 48 items targeted each domain. The facets represented the theoretical considerations of the authors. The questionnaire soon became widely accepted [113] but an even shorter version was requested by many researchers. This need was satisfied by publishing the NEO-FFI, which allows the user to assess the domains but not the facets. Each domain can be assessed with 12 items. Thus, only one fourth of the original items remained in the shortened version. Obviously, this can result in a loss of some substance. When selecting the items, the authors primarily chose items with strong loadings on their respective domains. This selection strategy did not directly aim to mirror the facet structure of the NEO-PI-R within the NEO-FFI [114]. For example, nine out of the 12 items for Extraversion as well as for Openness represent only three of the six facets. In the case of Extraversion, the main part of the domain for the NEO-FFI is built by Positive Emotions, Activity and Gregariousness. The other facets (i.e., Warmth, Assertiveness and Excitement-seeking) are represented by only one item each. The foundation of Openness in the NEO-FFI is made up of Ideas, Aesthetics and Values. Fantasy, Feelings and Actions are tapped by only one item each. Clearly, the underrepresentation of half of the facets of Extraversion and Openness may affect the relationship with other constructs and also between the two. For instance, their correlation is moderate for the German NEO-PI-R norm sample (*r* = 0.40) and remarkably low for the German NEO-FFI norm sample (*r* = 0.14). Thus, even seemingly harmless changes from a long to a short version of the same personality test can alter the content validity of its scores. It goes without saying that this also affects the interpretability of all higher order factors. 

Consequently, the present study should be replicated with different measurements ideally in a way so that the construct representation is comparable at all hierarchical levels. In addition, these measurements should assess specific personality traits (i.e., FFM facets as assessed in Study 1; or even below, see [115]) to investigate whether the combination of these specific personality traits with specific cognitive abilities (as assessed in Study 2) will reveal even stronger personality-intelligence correlations. Importantly, these tests should ensure a reliable measurement on the lowest hierarchical level. As seen in Table 1 and Table 3, the reliabilities were lowest for FFM facets and narrow abilities. This touches one of the underlying principles of the Brunswik symmetry (i.e., increased reliability through aggregation leads to larger correlations on higher levels [23]) and, thus, differences in reliabilities should be taken into account when personality-intelligence correlations are evaluated. In the present study, however, we found only negligible differences between disattenuated and uncorrected correlations (see Appendix A). Therefore, different personality-intelligence correlations at different aggregations levels could not be explained by differential reliabilities in our study. 

Although we applied broad operationalisations of reasoning (Study 1 and 2) and additional intelligence constructs (e.g., perceptual speed, memory, fluency; Study 2), the operationalisations did not cover the entire spectrum of intelligence (see e.g., Reference [94]) but rather focused on fluid intelligence. Most importantly, personality-intelligence relations based on crystallized intelligence were underrepresented in our investigation. Although Beauducel and Kersting [116] showed that fluency measurements as applied in our study are closely associated with crystallized intelligence, traditional knowledge tests as operationalisations of crystallized intelligence should be considered in future studies. This is even more important as previous research provided evidence that some personality traits are more strongly related to crystallized intelligence than to fluid intelligence (e.g., [41]). However, it should be noted that in most of the studies crystallized intelligence is often solely operationalized with verbal knowledge tests leading to a construct underrepresentation [117]. Or to put in another way, in terms of the Brunswik symmetry principle [23] verbal knowledge tests are lower level operationalisations of crystallized intelligence compared to tests that are balanced in content (i.e., based on verbal, numerical and figural tasks; see e.g., Reference [65]). As verbal intelligence showed the strongest correlation with personality in our study, it would be interesting for future studies to investigate whether the often reported higher correlation between personality and crystallized intelligence compared to fluid intelligence is driven by the overrepresentation of verbal stimuli in operationalisations of crystallized intelligence and the underrepresentation of verbal stimuli in operationalisations of fluid intelligence [117].

Furthermore, innovative measurements of personality and intelligence have the potential to increase our knowledge about the personality-intelligence relations. In recent years, there has been a trend towards using more fine-grained assessment of personality via ambulatory assessment, daily diary, or mobile sensing (e.g., [118]). With regard to the Brunswick principle, these approaches allow us to relate very specific parts of personality to intelligence. For instance, variance in behavioural indicators of personality could be related to more fine-grained cognitive processes (see below) and therefore add an additional level to the hierarchy in terms of the Brunswick principle. As some of the FFM facets were much more strongly related to intelligence than FFM dimensions, it is quite possible that personality and intelligence have the highest conceptual overlap on the process level. Thus, it might well be that even higher correlations will be found based on these fine-grained measurement approaches. 

A similar development is taking place regarding the measurement of intelligence. For example, interactive and dynamic tasks allow to study more fine-grained cognitive processes on the basis of log file analysis (e.g., exploration of problems [119,120,121]). These tasks are often labelled as measurements of complex problem solving (e.g., [122,123,124]) or dynamic decision making (e.g., [125,126]) but more and more evidence is provided that these tasks should be considered as innovative intelligence tests (e.g., [29,127,128]). Therefore, further insights about the association between intelligence and personality might be gained based on these fine-grained measurement approaches (e.g., higher levels of Conscientiousness may be associated with different exploration strategies in complex problems).

Finally, there are additional issues not addressed in the present study, which might be also important regarding personality-intelligence relations (see e.g., Reference [22]). First, we did not find systematic hints that non-linear relations would alter our results. This finding is in line with previous studies (e.g., [45,48,129]) that did not find evidence for curvilinear relations regarding personality, intelligence and external criteria (but see e.g., Reference [130]). Second, it might be worthwhile to investigate subgroups with regard to different personality-intelligence relations. Gender differences are regularly investigated in personality (e.g., [73]) and intelligence (e.g., [74,75]) research. In addition, considering less obviously classifiable groups might provide deeper insights regarding the personality-intelligence relations (e.g., based on latent class analysis). However, in order to conduct such analyses as in the present study or with regard to further research questions (e.g., subgroup analyses, non-linear relations), large sample sizes are needed to ensure a sufficiently large statistical power or precise estimation of the effects. As shown in the present study, the integrative data analysis (IDA) approach [56] is an ideal way to meet this requirement^8^. In addition, applying the IDA approach leads to potentially more heterogeneous and representative samples reducing the risk of attenuated correlations due to range restrictions—an issue that should be also considered when evaluating the results of the present study (i.e., most of the participants were high school or university students). On the other hand, such heterogeneous samples run the risk of unclear factor structures within personality measures (e.g., [134,135]). Some researchers have linked this phenomenon to differences in intelligence (also see [136]), while others see it as a consequence of differences in response bias (e.g., [137]).

## 5. Conclusions

The association between personality and intelligence is a complex issue. As demonstrated in the present paper, the Brunswik symmetry principle [23] can help to cope with the complexity. The advantage of taking the Brunswik symmetry principle into account is to potentially find large correlations that can be masked by asymmetrical comparisons. Therefore, we generally recommend applying broad operationalisations of personality and intelligence in order to analyse different hierarchical levels. Or to put it differently, the search for personality-intelligence relations will benefit most from elaborated measurements. 

## Figures and Tables

**Table 1:** Study 1: Descriptive statistics and reliability (McDonald’s Omega) (*N* = 682).

	*M*	*SD*	*Min*	*Max*	ω
Reasoning	63.72	9.98	28.33	88.89	0.90
Verbal Reasoning	63.94	10.99	27.12	89.83	0.75
Numerical Reasoning	69.84	15.62	23.33	100.00	0.90
Figural Reasoning	58.45	12.59	23.33	91.67	0.80
GFP	2.42	0.24	1.64	3.16	0.95
Stability	2.30	0.28	1.50	3.11	0.93
Plasticity	2.53	0.32	1.55	3.64	0.92
Openness	2.58	0.39	1.42	3.71	0.89
Conscientiousness	2.44	0.41	1.15	3.77	0.92
Extraversion	2.49	0.41	0.81	3.56	0.91
Agreeableness	2.40	0.37	0.98	3.38	0.89
Emotional Stability	2.07	0.48	0.67	3.42	0.93
O1: Fantasy	2.62	0.60	0.88	4.00	0.78
O2: Aesthetics	2.72	0.75	0.50	4.00	0.82
O3: Feelings	2.91	0.56	0.50	4.00	0.82
O4: Actions	2.19	0.49	0.88	3.62	0.60
O5: Ideas	2.55	0.67	0.50	4.00	0.82
O6: Values	2.46	0.45	1.25	3.88	0.54
C1: Competence	2.68	0.47	1.12	3.88	0.72
C2: Order	2.31	0.60	0.38	3.88	0.73
C3: Dutifulness	2.67	0.53	0.88	4.00	0.74
C4: Achievement Striving	2.50	0.52	1.12	3.88	0.71
C5: Self-Discipline	2.31	0.62	0.12	3.75	0.81
C6: Deliberation	2.14	0.62	0.25	3.88	0.80
E1: Warmth	2.91	0.51	0.50	4.00	0.77
E2: Gregariousness	2.56	0.64	0.25	4.00	0.80
E3: Assertiveness	2.11	0.66	0.25	3.75	0.82
E4: Activity	2.25	0.51	0.88	3.75	0.67
E5: Excitement-Seeking	2.30	0.62	0.50	3.88	0.62
E6: Positive Emotions	2.83	0.64	0.38	4.00	0.83
A1: Trust	2.41	0.56	0.38	3.88	0.77
A2: Straightforwardness	2.25	0.59	0.50	3.88	0.70
A3: Altruism	2.89	0.49	1.12	4.00	0.74
A4: Compliance	2.02	0.54	0.25	3.50	0.66
A5: Modesty	2.13	0.59	0.25	3.75	0.76
A6: Tender-Mindedness	2.67	0.44	1.00	3.75	0.64
-N1: Anxiety	1.91	0.70	0.00	3.75	0.82
-N2: Angry Hostility	2.20	0.60	0.25	3.75	0.74
-N3: Depression	2.30	0.76	0.25	4.00	0.86
-N4: Self-Consciousness	1.93	0.60	0.25	3.75	0.72
-N5: Impulsiveness	1.75	0.53	0.38	3.25	0.61
-N6: Vulnerability	2.32	0.61	0.12	3.75	0.81

**Table 2:** Study 1: Bootstrapped correlations (controlled for gender) between personality and intelligence. 95% CI in brackets.

	Reasoning		Verbal Reasoning		Numerical Reasoning		Figural Reasoning	
GFP	0.02	[−0.06, 0.10]	0.08	[0.01, 0.15]	−0.02	[−0.10, 0.06]	0.01	[−0.07, 0.08]
Stability	−0.02	[−0.09, 0.06]	0.02	[−0.05, 0.09]	−0.01	[−0.09, 0.06]	−0.05	[−0.12, 0.03]
Plasticity	0.05	[−0.03, 0.13]	0.10	[0.02, 0.18]	−0.02	[−0.09, 0.06]	0.05	[−0.03, 0.13]
Openness	0.22	[0.14, 0.29]	0.32	[0.25, 0.38]	0.07	[−0.01, 0.14]	0.15	[0.07, 0.23]
Conscientiousness	0.06	[−0.01, 0.13]	0.06	[−0.01, 0.13]	0.04	[−0.04, 0.11]	0.04	[−0.03, 0.11]
Extraversion	−0.12	[−0.20, −0.05]	−0.14	[−0.21, −0.07]	−0.09	[−0.17, −0.02]	−0.06	[−0.14, 0.01]
Agreeableness	−0.01	[−0.10, 0.07]	0.05	[−0.03, 0.12]	0.01	[−0.07, 0.09]	−0.08	[−0.17, −0.01]
Emotional Stability	−0.07	[−0.15, 0.00]	−0.05	[−0.12, 0.03]	−0.07	[−0.14, 0.01]	−0.05	[−0.13, 0.02]
O1: Fantasy	0.19	[0.12, 0.26]	0.20	[0.13, 0.27]	0.10	[0.03, 0.17]	0.17	[0.09, 0.24]
O2: Aesthetics	0.12	[0.05, 0.19]	0.18	[0.10, 0.25]	0.01	[−0.06, 0.08]	0.12	[0.04, 0.20]
O3: Feelings	0.12	[0.04, 0.20]	0.16	[0.08, 0.24]	0.06	[−0.02, 0.13]	0.08	[0.01, 0.17]
O4: Actions	0.00	[−0.08, 0.08]	0.09	[0.02, 0.16]	−0.03	[−0.11, 0.05]	−0.03	[−0.11, 0.05]
O5: Ideas	0.25	[0.17, 0.32]	0.31	[0.24, 0.38]	0.11	[0.04, 0.19]	0.19	[0.11, 0.26]
O6: Values	0.14	[0.06, 0.21]	0.33	[0.27, 0.40]	0.02	[−0.05, 0.10]	0.02	[−0.06, 0.09]
C1: Competence	0.07	[−0.00, 0.15]	0.13	[0.06, 0.20]	0.02	[−0.06, 0.10]	0.03	[−0.04, 0.11]
C2: Order	0.07	[−0.01, 0.14]	−0.00	[−0.07, 0.07]	0.04	[−0.03, 0.12]	0.10	[0.03, 0.18]
C3: Dutifulness	0.03	[−0.05, 0.10]	0.08	[0.01, 0.14]	0.01	[−0.06, 0.09]	−0.02	[−0.10, 0.06]
C4: Achievement Striving	−0.04	[−0.12, 0.04]	0.01	[−0.07, 0.08]	−0.08	[−0.15, 0.00]	−0.01	[−0.09, 0.07]
C5: Self-discipline	−0.00	[−0.07, 0.07]	−0.01	[−0.08, 0.06]	0.01	[−0.06, 0.08]	−0.01	[−0.08, 0.06]
C6: Deliberation	0.13	[0.05, 0.20]	0.08	[0.01, 0.15]	0.13	[0.05, 0.21]	0.08	[−0.00, 0.15]
E1: Warmth	−0.11	[−0.19, −0.03]	−0.09	[−0.16, −0.02]	−0.10	[−0.18, −0.02]	−0.07	[−0.15, 0.01]
E2: Gregariousness	−0.14	[−0.21, −0.07]	−0.13	[−0.20, −0.05]	−0.11	[−0.19, −0.04]	−0.09	[−0.16, −0.02]
E3: Assertiveness	−0.05	[−0.13, 0.02]	−0.05	[−0.12, 0.03]	−0.06	[−0.13, 0.01]	−0.01	[−0.09, 0.07]
E4: Activity	−0.04	[−0.12, 0.03]	−0.06	[−0.14, 0.01]	−0.02	[−0.10, 0.06]	−0.03	[−0.10, 0.05]
E5: Excitement-Seeking	−0.11	[−0.18, −0.03]	−0.21	[−0.28, −0.14]	−0.04	[−0.12, 0.03]	−0.02	[−0.10, 0.05]
E6: Positive Emotions	−0.05	[−0.13, 0.02]	−0.05	[−0.12, 0.02]	−0.04	[−0.12, 0.04]	−0.03	[−0.11, 0.04]
A1: Trust	0.02	[−0.06, 0.09]	0.12	[0.05, 0.19]	−0.01	[−0.09, 0.07]	−0.05	[−0.13, 0.02]
A2: Straightforwardness	0.07	[−0.01, 0.14]	0.07	[−0.00, 0.14]	0.09	[0.02, 0.16]	−0.01	[−0.08, 0.07]
A3: Altruism	−0.03	[−0.12, 0.05]	0.00	[−0.08, 0.08]	−0.04	[−0.12, 0.04]	−0.04	[−0.12, 0.04]
A4: Compliance	−0.02	[−0.10, 0.06]	0.04	[−0.04, 0.12]	0.01	[−0.07, 0.09]	−0.09	[−0.16, −0.01]
A5: Modesty	−0.09	[−0.17, −0.02]	−0.08	[−0.16, −0.01]	−0.02	[−0.10, 0.05]	−0.12	[−0.19, −0.04]
A6: Tender-Mindedness	0.01	[−0.07, 0.09]	0.04	[−0.03, 0.12]	0.02	[−0.06, 0.09]	−0.03	[−0.11, 0.05]
-N1: Anxiety	−0.03	[−0.11, 0.04]	−0.02	[−0.10, 0.05]	−0.03	[−0.10, 0.04]	−0.03	[−0.11, 0.05]
-N2: Angry Hostility	−0.01	[−0.09, 0.07]	0.04	[−0.03, 0.11]	−0.01	[−0.09, 0.07]	−0.05	[−0.13, 0.03]
-N3: Depression	−0.09	[−0.17, −0.02]	−0.06	[−0.13, 0.02]	−0.09	[−0.17, −0.02]	−0.06	[−0.13, 0.01]
-N4: Self-Consciousness	−0.11	[−0.19, −0.04]	−0.11	[−0.18, −0.03]	−0.12	[−0.18, −0.05]	−0.04	[−0.11, 0.04]
-N5: Impulsiveness	−0.01	[−0.09, 0.07]	−0.03	[−0.10, 0.05]	0.01	[−0.07, 0.08]	−0.01	[−0.08, 0.06]
-N6: Vulnerability	−0.06	[−0.13, 0.02]	−0.05	[−0.12, 0.02]	−0.04	[−0.12, 0.03]	−0.04	[−0.12, 0.03]

**Table 3:** Study 2: Descriptive statistics and reliability (McDonald’s Omega) (*N* = 413).

	*M*	*SD*	*Min*	*Max*	ω
*g*	47.24	7.88	16.59	72.47	0.90
Reasoning	49.50	13.67	10.97	90.68	0.86
Fluency	31.55	7.51	11.47	64.14	0.85
Speed	53.89	10.18	16.66	86.62	0.79
Memory	54.02	10.17	27.27	99.18	0.71
Verbal Intelligence	46.17	8.67	12.08	69.15	0.80
Numerical Intelligence	47.18	11.09	15.12	76.85	0.84
Figural Intelligence	46.37	8.94	19.42	73.59	0.77
Verbal Reasoning	52.99	15.21	11.94	96.11	0.74
Numerical Reasoning	53.28	17.30	9.17	95.56	0.75
Figural Reasoning	42.96	17.03	2.50	93.50	0.72
Verbal Fluency	25.74	8.25	3.85	58.01	0.79
Numerical Fluency	33.79	10.07	10.95	79.01	0.66
Figural Fluency	35.11	9.01	11.98	64.27	0.70
Verbal Speed	56.47	12.88	15.04	96.18	0.72
Numerical Speed	51.37	15.54	8.85	96.00	0.72
Figural Speed	53.85	10.83	26.09	100.00	0.69
Verbal Memory	51.75	11.82	16.21	100.00	0.53
Numerical Memory	50.68	15.15	12.73	100.00	0.62
Figural Memory	59.62	12.63	27.72	97.53	0.55
GFP	2.49	0.32	1.11	3.42	0.87
Stability	2.57	0.39	1.06	3.64	0.85
Plasticity	2.40	0.40	1.17	3.50	0.77
Openness	2.33	0.58	0.92	3.92	0.78
Conscientiousness	2.65	0.62	0.00	4.00	0.87
Extraversion	2.48	0.51	0.17	3.67	0.79
Agreeableness	2.63	0.47	0.75	3.92	0.74
Emotional Stability	2.43	0.60	0.25	3.82	0.84

**Table 4:** Study 2: Bootstrapped correlations (controlled for gender) between personality and intelligence. 95% CI in brackets.

	GFP		Stability		Plasticity		Openness		Conscientiousness		Extraversion		Agreeableness		Emotional Stability	
*g*	0.15	[0.05, 0.25]	0.14	[0.05, 0.24]	0.10	[0.00, 0.20]	0.06	[−0.04, 0.15]	0.13	[0.05, 0.22]	0.08	[−0.01, 0.18]	0.06	[−0.04, 0.15]	0.09	[−0.00, 0.18]
Reasoning	0.15	[0.04, 0.26]	0.09	[−0.01, 0.19]	0.16	[0.05, 0.26]	0.22	[0.12, 0.31]	0.07	[−0.02, 0.17]	−0.00	[−0.09, 0.10]	0.07	[−0.04, 0.17]	0.05	[−0.06, 0.14]
Fluency	0.13	[0.04, 0.22]	0.12	[0.02, 0.21]	0.08	[−0.01, 0.18]	0.01	[−0.09, 0.12]	0.12	[0.03, 0.20]	0.12	[0.03, 0.21]	0.02	[−0.07, 0.11]	0.10	[0.01, 0.18]
Speed	0.05	[−0.04, 0.15]	0.09	[0.00, 0.19]	−0.01	[−0.10, 0.08]	−0.09	[−0.18, 0.01]	0.11	[0.02, 0.20]	0.08	[−0.02, 0.18]	0.00	[−0.09, 0.09]	0.07	[−0.03, 0.16]
Memory	0.11	[0.02, 0.21]	0.14	[0.05, 0.24]	0.04	[−0.05, 0.14]	−0.02	[−0.12, 0.08]	0.12	[0.04, 0.21]	0.08	[−0.01, 0.19]	0.08	[−0.02, 0.18]	0.09	[−0.00, 0.17]
verbal Intelligence	0.15	[0.04, 0.24]	0.06	[−0.03, 0.16]	0.18	[0.09, 0.27]	0.21	[0.11, 0.29]	0.07	[−0.03, 0.16]	0.04	[−0.05, 0.14]	0.01	[−0.08, 0.11]	0.03	[−0.06, 0.12]
numerical Intelligence	0.11	[0.00, 0.22]	0.19	[0.09, 0.29]	−0.01	[−0.11, 0.10]	−0.07	[−0.17, 0.03]	0.18	[0.09, 0.27]	0.06	[−0.04, 0.16]	0.09	[−0.02, 0.20]	0.12	[0.02, 0.21]
figural Intelligence	0.14	[0.04, 0.24]	0.08	[−0.02, 0.17]	0.15	[0.06, 0.24]	0.14	[0.04, 0.24]	0.05	[−0.04, 0.14]	0.08	[−0.02, 0.18]	0.04	[−0.05, 0.14]	0.07	[−0.03, 0.15]
verbal Reasoning	0.15	[0.05, 0.24]	0.03	[−0.08, 0.13]	0.21	[0.12, 0.31]	0.31	[0.21, 0.39]	0.03	[−0.07, 0.13]	−0.02	[−0.10, 0.08]	0.01	[−0.09, 0.11]	0.01	[−0.09, 0.11]
numerical Reasoning	0.14	[0.04, 0.24]	0.17	[0.07, 0.26]	0.05	[−0.06, 0.16]	0.07	[−0.04, 0.17]	0.16	[0.07, 0.24]	0.00	[−0.09, 0.09]	0.11	[0.01, 0.21]	0.09	[−0.02, 0.19]
figural Reasoning	0.09	[−0.01, 0.19]	0.01	[−0.09, 0.10]	0.14	[0.03, 0.24]	0.18	[0.08, 0.28]	−0.03	[−0.13, 0.06]	0.01	[−0.08, 0.10]	0.04	[−0.05, 0.14]	0.01	[−0.09, 0.11]
verbal Fluency	0.10	[0.02, 0.17]	0.03	[−0.05, 0.11]	0.12	[0.04, 0.20]	0.04	[−0.05, 0.13]	0.03	[−0.06, 0.12]	0.14	[0.06, 0.22]	0.01	[−0.08, 0.10]	0.03	[−0.05, 0.11]
numerical Fluency	0.04	[−0.05, 0.14]	0.12	[0.01, 0.22]	−0.05	[−0.15, 0.05]	−0.11	[−0.20, −0.00]	0.12	[0.03, 0.21]	0.05	[−0.04, 0.15]	0.01	[−0.09, 0.11]	0.10	[−0.00, 0.20]
figural Fluency	0.18	[0.08, 0.27]	0.13	[0.04, 0.22]	0.16	[0.06, 0.25]	0.11	[0.01, 0.22]	0.13	[0.04, 0.22]	0.11	[0.02, 0.21]	0.03	[−0.06, 0.11]	0.11	[0.02, 0.20]
verbal Speed	0.05	[−0.04, 0.15]	0.04	[−0.06, 0.14]	0.04	[−0.06, 0.14]	0.04	[−0.05, 0.14]	0.06	[−0.04, 0.16]	0.01	[−0.08, 0.10]	−0.02	[−0.12, 0.08]	0.04	[−0.05, 0.14]
numerical Speed	0.05	[−0.04, 0.15]	0.11	[0.02, 0.21]	−0.03	[−0.13, 0.06]	−0.10	[−0.19, −0.00]	0.13	[0.03, 0.22]	0.06	[−0.03, 0.15]	0.04	[−0.05, 0.13]	0.06	[−0.04, 0.16]
figural Speed	0.01	[−0.08, 0.10]	0.05	[−0.04, 0.14]	−0.04	[−0.13, 0.06]	−0.17	[−0.25, −0.08]	0.06	[−0.03, 0.14]	0.13	[0.04, 0.22]	−0.02	[−0.11, 0.07]	0.07	[−0.03, 0.15]
verbal Memory	0.08	[−0.01, 0.17]	0.08	[−0.01, 0.17]	0.04	[−0.05, 0.14]	0.01	[−0.09, 0.11]	0.09	[0.00, 0.18]	0.05	[−0.03, 0.13]	0.05	[−0.05, 0.14]	0.03	[−0.06, 0.12]
numerical Memory	0.05	[−0.06, 0.16]	0.15	[0.04, 0.25]	−0.06	[−0.17, 0.04]	−0.16	[−0.25, −0.07]	0.12	[0.03, 0.20]	0.09	[−0.02, 0.20]	0.08	[−0.03, 0.19]	0.10	[0.01, 0.19]
figural Memory	0.13	[0.04, 0.24]	0.09	[−0.01, 0.18]	0.13	[0.03, 0.22]	0.13	[0.04, 0.23]	0.08	[−0.02, 0.16]	0.05	[−0.05, 0.14]	0.05	[−0.05, 0.15]	0.05	[−0.04, 0.14]

## Footnotes

1 From here on abbreviated as reasoning.

2 Sometimes the connection to the Brunswik symmetry principle [23] is not made explicitly in the literature. In addition, research considering the bandwidth-fidelity dilemma [40] is closely related to the Brunswik symmetry principle. In fact, the Brunswik symmetry principle as part of Wittmann’s [23] multivariate reliability theory can be considered as a formalization of the bandwidth-fidelity dilemma.

3 In [61] the same data set was used. In the original publication [58], only a subsample was used for the analysis as at one school testing time was more limited and students filled in a shortened testing battery not including the questionnaire on help-seeking. In the present study, we used the complete data set. Thus, the reported sample sizes differ.

4 Please note that Reeve et al. [48] argued that even |*r*_diff_|≥ 0.05 can be considered as substantial with regard to personality-intelligence relations. However, such differences would need enormously large sample sizes to ensure acceptable statistical power [69].

5 Please note that Wilcox’ [70] approach is based on Winsorized correlations (i.e., Pearson’s correlations with a correction for outliers).

6 Please note that the original conceptualization of the BIS model does not consider abilities at the lowest level as specific abilities but as performances based on a combination of second level abilities [1,93]. However, integrating commonly accepted and contemporary theories such as the Cattell-Horn-Carroll (CHC) theory [94] into the BIS model demonstrates that these performances are considered as specific abilities in other theories [95]. For the sake of compatibility between the different intelligence theories, we consider these performances as specific abilities in the present study.

7 Please note that we transformed the 45 test scores into POMP scores [96] instead of z-standardized scores as originally recommended in the test manual.

8 It should be emphasized that the IDA approach is dependent on the availability of data sets. During the preparation of the present study, we made similar experiences as, for example, Wicherts et al. [131] and Vanpaemel et al. [132] regarding the lack of available data from previously published studies. Therefore, we encourage researchers to follow the suggestions regarding reproducible science so that open data will be much more common for psychological research (e.g., [133]).

## Appendix A

Table A1 (Study 1) and Table A2 (Study 2) display the disattenuated correlations based on Padilla and Veprinsky’s [79] bootstrap approach. McDonalds’s omega was used as reliability estimation (see Table 1 for Study 1, Table 3 for Study 2). The differences between the uncorrected and corrected correlations were negligible for most of the correlations. Virtually no differences were found regarding the evaluation of the Brunswik symmetry principle when disattenuated correlations were considered instead of uncorrected correlations. Thus, the results based on disattenuated correlations are not presented.

**Table A1:** Study 1: Bootstrapped disattenuated correlations (controlled for gender). 95% CI in brackets.

	Reasoning		Verbal Reasoning		Numerical Reasoning		Figural Reasoning	
GFP	0.02	[−0.06, 0.11]	0.09	[0.01, 0.18]	−0.02	[−0.11, 0.06]	0.01	[−0.08, 0.10]
Stability	−0.02	[−0.10, 0.06]	0.03	[−0.06, 0.11]	−0.02	[−0.10, 0.07]	−0.05	[−0.14, 0.03]
Plasticity	0.06	[−0.03, 0.14]	0.12	[0.03, 0.21]	−0.02	[−0.10, 0.07]	0.06	[−0.03, 0.15]
Openness	0.24	[0.15, 0.32]	0.39	[0.30, 0.47]	0.08	[−0.01, 0.16]	0.18	[0.09, 0.27]
Conscientiousness	0.06	[−0.01, 0.14]	0.07	[−0.01, 0.16]	0.04	[−0.04, 0.12]	0.05	[−0.04, 0.13]
Extraversion	−0.14	[−0.22, −0.06]	−0.17	[−0.26, −0.08]	−0.10	[−0.19, −0.02]	−0.07	[−0.16, 0.01]
Agreeableness	−0.02	[−0.11, 0.07]	0.06	[−0.03, 0.14]	0.01	[−0.07, 0.10]	−0.10	[−0.20, −0.01]
Emotional Stability	−0.08	[−0.17, 0.00]	−0.06	[−0.15, 0.03]	−0.07	[−0.15, 0.01]	−0.06	[−0.15, 0.03]
O1: Fantasy	0.23	[0.15, 0.31]	0.26	[0.17, 0.35]	0.12	[0.04, 0.20]	0.21	[0.12, 0.31]
O2: Aesthetics	0.14	[0.06, 0.23]	0.23	[0.13, 0.32]	0.01	[−0.07, 0.09]	0.15	[0.05, 0.24]
O3: Feelings	0.14	[0.05, 0.24]	0.21	[0.11, 0.30]	0.07	[−0.03, 0.15]	0.10	[0.01, 0.20]
O4: Actions	0.00	[−0.11, 0.11]	0.13	[0.03, 0.23]	−0.05	[−0.16, 0.06]	−0.05	[−0.16, 0.07]
O5: Ideas	0.29	[0.20, 0.37]	0.39	[0.30, 0.48]	0.13	[0.04, 0.22]	0.23	[0.14, 0.32]
O6: Values	0.20	[0.09, 0.31]	0.52	[0.42, 0.62]	0.03	[−0.08, 0.14]	0.03	[−0.09, 0.14]
C1: Competence	0.09	[−0.00, 0.18]	0.18	[0.09, 0.27]	0.02	[−0.07, 0.12]	0.04	[−0.06, 0.14]
C2: Order	0.08	[−0.01, 0.17]	−0.00	[−0.10, 0.09]	0.05	[−0.04, 0.14]	0.14	[0.04, 0.23]
C3: Dutifulness	0.03	[−0.06, 0.13]	0.11	[0.01, 0.19]	0.02	[−0.08, 0.12]	−0.03	[−0.13, 0.07]
C4: Achievement Striving	−0.05	[−0.15, 0.05]	0.01	[−0.10, 0.12]	−0.10	[−0.19, 0.00]	−0.01	[−0.12, 0.09]
C5: Self-discipline	−0.00	[−0.08, 0.08]	−0.01	[−0.10, 0.08]	0.02	[−0.07, 0.10]	−0.01	[−0.10, 0.08]
C6: Deliberation	0.15	[0.06, 0.23]	0.10	[0.01, 0.20]	0.15	[0.06, 0.24]	0.09	[−0.00, 0.18]
E1: Warmth	−0.14	[−0.23, −0.04]	−0.12	[−0.21, −0.02]	−0.12	[−0.22, −0.03]	−0.09	[−0.19, 0.01]
E2: Gregariousness	−0.17	[−0.25, −0.08]	−0.16	[−0.26, −0.06]	−0.13	[−0.22, −0.05]	−0.11	[−0.20, −0.03]
E3: Assertiveness	−0.06	[−0.15, 0.03]	−0.06	[−0.15, 0.03]	−0.07	[−0.15, 0.01]	−0.02	[−0.12, 0.08]
E4: Activity	−0.06	[−0.16, 0.04]	−0.09	[−0.19, 0.02]	−0.02	[−0.12, 0.08]	−0.04	[−0.14, 0.07]
E5: Excitement-Seeking	−0.14	[−0.25, −0.05]	−0.30	[−0.41, −0.20]	−0.06	[−0.16, 0.04]	−0.03	[−0.14, 0.07]
E6: Positive Emotions	−0.06	[−0.16, 0.03]	−0.07	[−0.16, 0.03]	−0.05	[−0.14, 0.04]	−0.04	[−0.14, 0.05]
A1: Trust	0.02	[−0.08, 0.11]	0.16	[0.07, 0.25]	−0.02	[−0.11, 0.08]	−0.07	[−0.16, 0.03]
A2: Straightforwardness	0.09	[−0.01, 0.18]	0.09	[−0.01, 0.19]	0.12	[0.02, 0.20]	−0.01	[−0.11, 0.09]
A3: Altruism	−0.04	[−0.14, 0.06]	0.00	[−0.10, 0.11]	−0.05	[−0.14, 0.05]	−0.05	[−0.15, 0.05]
A4: Compliance	−0.03	[−0.13, 0.08]	0.05	[−0.06, 0.17]	0.01	[−0.09, 0.11]	−0.12	[−0.22, −0.01]
A5: Modesty	−0.11	[−0.20, −0.02]	−0.11	[−0.21, −0.01]	−0.03	[−0.12, 0.06]	−0.15	[−0.25, −0.06]
A6: Tender-Mindedness	0.01	[−0.09, 0.12]	0.06	[−0.05, 0.17]	0.02	[−0.08, 0.12]	−0.05	[−0.16, 0.06]
-N1: Anxiety	−0.04	[−0.13, 0.05]	−0.02	[−0.12, 0.07]	−0.03	[−0.12, 0.05]	−0.04	[−0.13, 0.06]
-N2: Angry Hostility	−0.02	[−0.11, 0.08]	0.06	[−0.04, 0.15]	−0.02	[−0.11, 0.08]	−0.07	[−0.17, 0.04]
-N3: Depression	−0.11	[−0.19, −0.02]	−0.07	[−0.16, 0.02]	−0.11	[−0.19, −0.03]	−0.07	[−0.16, 0.02]
-N4: Self-Consciousness	−0.14	[−0.23, −0.05]	−0.15	[−0.25, −0.04]	−0.14	[−0.22, −0.06]	−0.05	[−0.15, 0.05]
-N5: Impulsiveness	−0.01	[−0.12, 0.09]	−0.04	[−0.15, 0.07]	0.01	[−0.09, 0.11]	−0.01	[−0.12, 0.09]
-N6: Vulnerability	−0.07	[−0.16, 0.02]	−0.07	[−0.16, 0.02]	−0.05	[−0.14, 0.03]	−0.05	[−0.14, 0.04]

**Table A2:** Study 2: Bootstrapped disattenuated correlations (controlled for gender). 95% CI in brackets.

	GFP		Stability		Plasticity		Openness		Conscientiousness		Extraversion		Agreeableness		Emotional Stability	
*g*	0.17	[0.05, 0.28]	0.16	[0.05, 0.27]	0.12	[0.00, 0.24]	0.07	[−0.05, 0.18]	0.15	[0.05, 0.25]	0.10	[−0.02, 0.21]	0.07	[−0.05, 0.19]	0.11	[−0.00, 0.21]
Reasoning	0.18	[0.05, 0.30]	0.10	[−0.01, 0.22]	0.20	[0.07, 0.32]	0.26	[0.15, 0.37]	0.08	[−0.03, 0.20]	−0.00	[−0.11, 0.12]	0.08	[−0.05, 0.21]	0.06	[−0.07, 0.16]
Fluency	0.15	[0.04, 0.25]	0.14	[0.03, 0.24]	0.10	[−0.01, 0.23]	0.01	[−0.11, 0.14]	0.13	[0.03, 0.24]	0.14	[0.03, 0.25]	0.02	[−0.09, 0.14]	0.12	[0.01, 0.22]
Speed	0.06	[−0.05, 0.18]	0.11	[0.00, 0.24]	−0.02	[−0.12, 0.10]	−0.11	[−0.23, 0.01]	0.13	[0.03, 0.24]	0.10	[−0.02, 0.23]	0.00	[−0.11, 0.12]	0.09	[−0.03, 0.20]
Memory	0.14	[0.02, 0.27]	0.18	[0.07, 0.30]	0.05	[−0.07, 0.19]	−0.03	[−0.16, 0.11]	0.16	[0.05, 0.27]	0.11	[−0.01, 0.25]	0.11	[−0.02, 0.25]	0.11	[−0.00, 0.22]
verbal Intelligence	0.18	[0.05, 0.29]	0.07	[−0.03, 0.19]	0.23	[0.11, 0.35]	0.26	[0.14, 0.37]	0.08	[−0.04, 0.20]	0.05	[−0.06, 0.18]	0.02	[−0.10, 0.14]	0.04	[−0.08, 0.15]
numerical Intelligence	0.13	[0.00, 0.25]	0.22	[0.10, 0.34]	−0.02	[−0.14, 0.12]	−0.08	[−0.21, 0.04]	0.21	[0.10, 0.32]	0.07	[−0.04, 0.19]	0.11	[−0.02, 0.25]	0.14	[0.02, 0.25]
figural Intelligence	0.18	[0.05, 0.30]	0.10	[−0.02, 0.21]	0.20	[0.08, 0.31]	0.18	[0.05, 0.30]	0.06	[−0.04, 0.18]	0.10	[−0.02, 0.24]	0.06	[−0.06, 0.18]	0.08	[−0.03, 0.19]
verbal Reasoning	0.19	[0.06, 0.30]	0.03	[−0.10, 0.16]	0.28	[0.16, 0.42]	0.40	[0.27, 0.52]	0.04	[−0.09, 0.16]	−0.02	[−0.14, 0.10]	0.02	[−0.12, 0.15]	0.01	[−0.12, 0.14]
numerical Reasoning	0.17	[0.04, 0.30]	0.21	[0.09, 0.33]	0.07	[−0.07, 0.21]	0.09	[−0.05, 0.22]	0.19	[0.08, 0.30]	0.00	[−0.11, 0.12]	0.14	[0.01, 0.28]	0.11	[−0.02, 0.24]
figural Reasoning	0.11	[−0.01, 0.24]	0.01	[−0.11, 0.13]	0.18	[0.05, 0.32]	0.24	[0.10, 0.37]	−0.03	[−0.16, 0.08]	0.01	[−0.11, 0.13]	0.06	[−0.07, 0.19]	0.01	[−0.12, 0.14]
verbal Fluency	0.12	[0.03, 0.21]	0.04	[−0.06, 0.14]	0.16	[0.05, 0.26]	0.06	[−0.06, 0.17]	0.04	[−0.07, 0.14]	0.18	[0.08, 0.28]	0.01	[−0.11, 0.14]	0.04	[−0.07, 0.14]
numerical Fluency	0.06	[−0.07, 0.19]	0.16	[0.01, 0.29]	−0.07	[−0.21, 0.08]	−0.15	[−0.28, −0.00]	0.16	[0.04, 0.28]	0.07	[−0.06, 0.20]	0.01	[−0.13, 0.15]	0.14	[−0.00, 0.27]
figural Fluency	0.23	[0.11, 0.35]	0.17	[0.05, 0.29]	0.21	[0.08, 0.34]	0.15	[0.01, 0.29]	0.16	[0.05, 0.29]	0.15	[0.02, 0.28]	0.04	[−0.08, 0.15]	0.14	[0.02, 0.26]
verbal Speed	0.07	[−0.05, 0.19]	0.06	[−0.07, 0.18]	0.05	[−0.08, 0.18]	0.06	[−0.06, 0.19]	0.08	[−0.05, 0.20]	0.02	[−0.11, 0.14]	−0.03	[−0.17, 0.11]	0.06	[−0.06, 0.18]
numerical Speed	0.06	[−0.05, 0.19]	0.14	[0.02, 0.26]	−0.04	[−0.17, 0.09]	−0.13	[−0.25, −0.00]	0.16	[0.04, 0.28]	0.08	[−0.04, 0.20]	0.05	[−0.07, 0.18]	0.08	[−0.05, 0.20]
figural Speed	0.01	[−0.11, 0.13]	0.07	[−0.06, 0.19]	−0.05	[−0.19, 0.08]	−0.23	[−0.34, −0.11]	0.07	[−0.04, 0.19]	0.18	[0.05, 0.30]	−0.03	[−0.16, 0.10]	0.09	[−0.03, 0.20]
verbal Memory	0.11	[−0.02, 0.26]	0.12	[−0.01, 0.26]	0.06	[−0.08, 0.22]	0.02	[−0.13, 0.17]	0.14	[0.00, 0.26]	0.07	[−0.05, 0.21]	0.08	[−0.07, 0.23]	0.05	[−0.09, 0.18]
numerical Memory	0.07	[−0.07, 0.22]	0.20	[0.06, 0.34]	−0.09	[−0.24, 0.06]	−0.23	[−0.36, −0.10]	0.16	[0.04, 0.27]	0.13	[−0.03, 0.28]	0.12	[−0.04, 0.28]	0.14	[0.01, 0.26]
figural Memory	0.19	[0.05, 0.35]	0.13	[−0.02, 0.26]	0.20	[0.05, 0.34]	0.20	[0.06, 0.35]	0.11	[−0.03, 0.24]	0.07	[−0.07, 0.21]	0.08	[−0.08, 0.23]	0.08	[−0.06, 0.20]
